# Genetic histories of individuals from multi-faith medieval Sicily

**DOI:** 10.1371/journal.pone.0350298

**Published:** 2026-06-24

**Authors:** Aurore Monnereau, Paola Orecchioni, Derek Hamilton, Eleanor Joan Green, Ian Noble, Richard Hagan, Marcela Sandoval-Velasco, Alessandra Molinari, Martin Carver, Camilla F. Speller, Nathan Wales

**Affiliations:** 1 Department of Archaeology, University of York, York, United Kingdom; 2 Dipartimento di Storia, Patrimonio Culturale, Formazione e Società, Università degli Studi di Roma Tor Vergata, Rome, Italy; 3 SUERC, University of Glasgow, Glasgow, United Kingdom; 4 Center for Genomic Sciences, National Autonomous University of Mexico, Cuernavaca, Mexico; 5 The GLOBE Institute, Faculty of Health and Medical Sciences, University of Copenhagen, Copenhagen, Denmark; 6 Department of Anthropology, University of British Columbia, Vancouver, Canada; Københavns Universitet: Kobenhavns Universitet, DENMARK

## Abstract

Medieval Sicily, located at the intersection of Europe, North Africa and the Near East, experienced successive political and religious transitions under Byzantine, Aghlabid, Fatimid, Norman, and Swabian rule. While it is well established that these events led to multi-faith societies, the long-term genetic impact of regime change is unclear. To evaluate this potential impact, we applied ancient genomic analysis to 111 archaeological Sicilian individuals, leading to successful mitochondrial haplotype inferences for 67 individuals and genome-wide analyses for 32 individuals dated between the 5th and 15th centuries CE. In contrast to simple narratives of population replacement, the data indicate nuanced and unappreciated demographic shifts. Several individuals dating before the Islamic conquest of Sicily exhibit substantial North African ancestry, evidencing movement across the Mediterranean Sea before this conquest. Individuals buried in Islamic cemeteries during the 9th to 11th century were found to have diverse ancestries deriving from populations around the Mediterranean Basin, however, the same ancestry components are also found in earlier periods, limiting what can be inferred about intra-Mediterranean migrations in this dataset. Nonetheless, the Islamic period marks the appearance of individuals with distant ancestral origins, West Africa and Northern Europe. During the Norman period, Christian and Islamic burials show the same genetic diversity maintained for hundreds of years, however, by the late medieval period, the ancestry components shifted toward modern European populations. Altogether, the study demonstrates the value of examining recent periods with ancient DNA methodologies to better understand how culture, identity and mobility impacted demography in the past.

## Introduction

Between the 5th and 15th centuries CE, the Mediterranean world and its surrounding regions experienced profound transformations, including the fragmentation of the Western Roman Empire, the expansion of Islamic politics, the military and religious mobilisations of the Crusades, and recurrent demographic crises such as the Black Death. Within this wider context, medieval Sicily occupied a position of exceptional strategic and economic importance. Its fertile plains and central location within the Mediterranean basin made it a critical hub in the networks of trade, conflict, and cultural transmission that connected Latin Christendom, the Islamic world, and the Byzantine East. As a result, the island became the target of successive powers, including the Byzantine Empire, Arab emirates, Norman rulers, the Hohenstaufen, and the Crown of Aragon. Each of them was seeking to control its ports and influence over the central Mediterranean. Following the fall of the Roman Empire and then decades of control by Vandals and Goths [1], Sicily was governed by Byzantines from 535–827 CE [2], by Sunni and Shıʿa Muslims in the 9th and 10th centuries [3], and finally by Christian dynasties [4, 5]. While historical sources chronicle Sicily’s battles and coronations, less information is available to track the impact of medieval regime change on local populations. Although some sources indicate a prosperous, multifaith community, the degree of social integration remains unclear. For example, the Muslim traveller Ibn Hawqal implied interfaith marriage between Muslims and Christian in 973 [3, 6] while in 1184 Ibn Jubayr reported tolerance and shared fashions in Palermo, although the faith groups occupied different quarters of the city [6]. The limited demographic data for medieval Sicily hinders a detailed understanding of population mobility and the social effects of political transitions.

Ancient DNA (aDNA) research, or “palaeogenomics”, provides an avenue to examine past peoples’ kinship, genetic affiliations and admixture with other groups [7]. While often applied to prehistoric periods [e.g., 8], DNA from individuals excavated from more recent contexts allows for syntheses with historic documents to challenge preconceptions and go beyond what documents record. For Sicily, Reitsema et al. [9] demonstrated the value of aDNA from historic periods at the Greek colony of Himera on Sicily’s north coast. Through genome-wide sequencing, the researchers found 5th century BCE Greek soldiers (480 BCE), were genetically distinct from the civilian people and the Greek soldiers in 409 BCE at Himera, showing links to distant regions, including the Balkans, the Baltics, the Caucasus and the Eurasian Steppe. The diverse ancestry of the Greek army suggests mercenaries moved long distances, in a manner that has not been fully recognized for Himera from written sources.

For medieval Sicily, genomic data has so far only been reported for one early medieval individual from Lilybaeum [10] and 21 individuals buried during the 11–13th centuries at the rural site of Segesta [11]. The latter study, undertaken by our team with similar methods, focused on people interred in adjacent Islamic and Christian cemeteries which were likely used contemporaneously for a period of time. Ancestry analyses indicated that the faith communities at Segesta had distinct genetic backgrounds, with most individuals buried in the Islamic cemetery showing affinity to modern populations from southern Europe, southeastern Europe, the Near East and towards North Africa, while individuals from the Christian cemetery grouped with modern populations from eastern, southern, southeastern and western Europe. None of the analysed individuals displayed admixture between the groups, but there was one genetic outlier: a biologically male individual from the Islamic cemetery with exclusively sub-Saharan African ancestry. While the findings at Segesta provide a novel perspective on life in medieval villages, one cannot assume they are representative of the wider patterns of medieval Sicily, as the genetic nuclear analyses were focused on 15 individuals from a single location.

**Fig 1:**
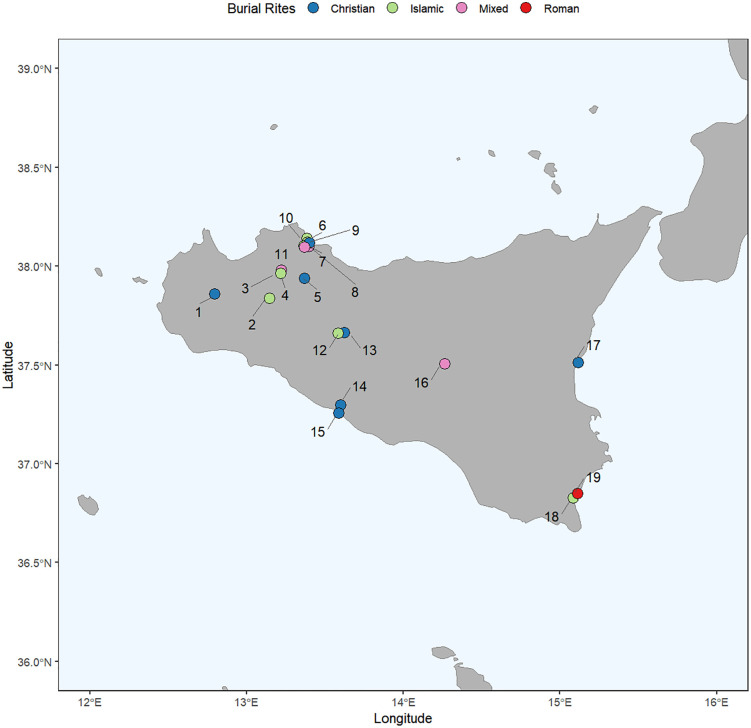
Locations of sample collection sites in this study. 1: San Miceli (Salemi), 2: Monte Maranfusa, 3: Monte Iato, 4: Monte Iato/Castellazzo, 5: Contrada Sant’Agata (Piana degli Albanesi), 6: Palermo, Castello San Pietro, 7: Palermo, La Gancia, 8: Palermo, Oratorio dei Bianchi, 9: Palermo, Palazzo Abatellis, 10: Palermo, Via Guardione, 11: Palermo, Corso dei Mille, 12: Castronovo/Colle San Vitale, 13: Casale San Pietro (Castronovo di Sicilia), 14: Agrigento, 15: San Leone (Agrigento), 16: Enna, 17: Catania, Sant’Agata La Vetere, 18: Villa del Tellaro, 19: Villa del Tellaro (Roman). Map created in R [12] using public domain data and ggplot2 [13], sf [14], ggrepel [15], rnaturalearth [16] and rnaturalearthdata [17] packages.

To more fully explore the impact of regime change on populations across space and time in medieval Sicily, we applied the radiocarbon and aDNA methodology utilised at the site of Segesta to 111 additional individuals from 18 archaeological sites spread across Sicily and dating from the Roman to Late Middle Ages (Fig 1). By evaluating the ancestry patterns between burial rites and within phases of the Middle Ages, we reach new perspectives on past group diversity and migrations to Sicily, find evidence of population turnovers associated with regime changes, and better appreciate when the ancestry of modern Sicilians began to crystalize.

## Results

### Chronological framework

Radiocarbon dating was performed using accelerator mass spectrometry for 91 of 111 individuals, targeting skeletal elements that demonstrated presence of DNA (S1 File). As the marine reservoir effect (MRE) can lead to erroneously old dates for individuals with diets rich in marine resources [18], isotopic values for δ^15^N and δ^13^C were also measured on the skeletal element. Following calibration and correction for the MRE, individuals were determined to date as early as 110 BCE–115 CE and as late as 1690–1935 CE (2σ is provided for all calibrated dates, i.e., 95.4% confidence interval). This range of calibrated dates stretches from the Roman period to after the end of Swabian rule of Sicily, thereby providing genetic information preceding the medieval period to the beginning of the modern era.

**Fig 2:**
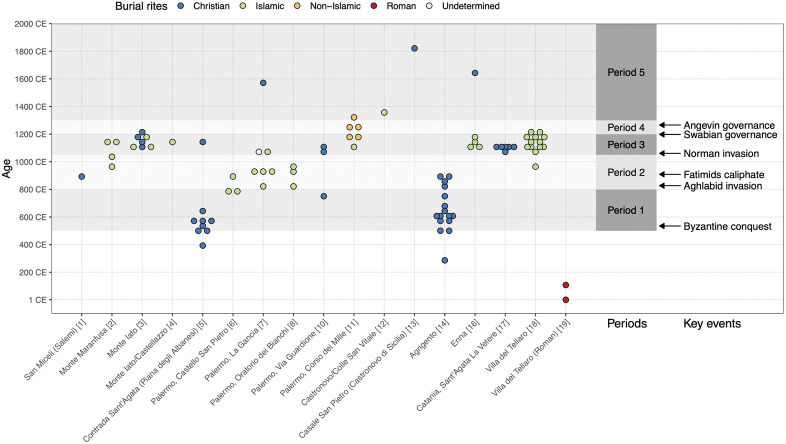
Chronological distribution of individuals tested, listed by site and presented with associated periods and key historical dates: 535 CE Byzantine conquest of Sicily, 827 CE Aghlabid invasion of Sicily, 909 CE Start of Fātimids caliphate, 1061 CE Norman invasion of Sicily, 1198 Swabian governance, and 1266 CE Angevin kings control Sicily. Sites are ordered in a largely west-to-east continuum, as shown in Fig 1. The age of each individual is displayed as the mean age of the calibrated radiocarbon measurement, as calculated by OxCal. Individual data points were arranged using the beeswarm package [20] for ggplot2 [13] to improve visualisation. Note that Palazzo Abatellis (listed as the ninth site in Fig 1) is not shown as it had poor DNA preservation and no direct AMS dates.

Due to plateaux and reversals in the radiocarbon curve, many calibrated dates cannot be unambiguously associated with one political regime. Rather, age ranges of some individuals span several centuries, such as individual GABN5 buried at the site of La Gancia in west Palermo, whose calibrated date of 685–970 CE overlaps with rule by the Byzantines, the Aghlabid dynasty (Sunni Muslim), and the Fatimid Caliphate (Shıʿa Muslim). To accommodate the limits of the wide age estimates, we present the genetic data using the mean age of the calibrated radiocarbon date corrected for the MRE (Fig 2) using this simplified chronological framework of five major periods of medieval Sicily:

- Period 1: 500–800 CE, largely corresponding to Byzantine rule
- Period 2: 800–1050 CE, the phase of Islamic governance
- Period 3: 1050–1200 CE, largely the period of Norman rule
- Period 4: 1200–1300 CE, the time of Swabian governance
- Period 5: after 1300 CE, end of Angevin control, terminal- and post-medieval period

Individuals are cautiously associated with these periods using the mean age of the calibrated date, as calculated by OxCal [19]. While these mean ages of the individual are distributed across the defined periods (Before Period 1: n = 7, Period 1: n = 21; Period 2: n = 16; Period 3: n = 37; Period 4: n = 5; Period 5: n = 5), it is important to reiterate that the full radiocarbon ranges of 77 individuals overlap with at least two of the defined periods. Nonetheless, this approach helps provide a general sequence for the individuals in the dataset and a way to explore broad temporal patterns in genetic change in medieval Sicily.

### Authenticated DNA

Following the implementation of aDNA methodologies to extract DNA from archaeological bone [21] and convert it to Illumina libraries [22, 23] (S1 File), shotgun sequencing demonstrated highly variable levels of preservation, with endogenous content ranging from <0.01 to 79.01% (mean = 15.42%) (S1 Table). Libraries with higher endogenous content were preferentially selected for deeper sequencing, achieving depth of coverage (DoC) on the nuclear genome ranging from <0.01 (the lowest is GABN7 at 0.00000047✕) to 2.54✕ (mean 0.34✕). Deamination patterns were observed in our dataset (S1 File, S1 Table). Of the 111 individuals screened for DNA content, 71 yielded at least around 100,000 sequencing reads that mapped to the human reference genome, a threshold that has been suggested to reliably characterize genetic sex of an individual [24]. Thus, among those, the analysis showed (S1 File, S1 Table): 33 XX individuals (separately counting QERBN6 and QERBN28, although these remains may originate from a single individual), 29 XY individuals, 3 individuals consistent with XY but not XX and one unassigned individual (QERBN20, whose ratio of X to Y sequences fell just below 0.075, the cutoff for XY individuals). The results for all archaeological individuals, including those with fewer reads than Skoglund et al.’s [24] recommended threshold are reported (S1 File, S1 Table); however; those below the cutoff should be interpreted with caution.

To augment the whole genome DNA sequencing data, in-solution enrichment of mitochondrial DNA was performed on libraries with low endogenous content. For medieval individuals with sufficient data for analysis, consistently low levels of human contamination were observed (S1 File, S1 Table): Schmutzi software [25] found the mitochondrial sequences of 64 individuals had < 5% contamination and ANGSD [26] calculated that X-chromosome contamination in 17 XY individuals was < 0.5%. Mitochondrial sequences could be reliably characterised with Haplogrep software [27] in 67 individuals (>10✕ DoC), and a diverse set of mitochondrial macro-haplogroups were observed, including H1, H2, H3, H5, H7, I3, J1, J2, K1, L0, L2, L3, T1, T2, U1, U4, U5, U6, V, V1, W and X2. Y-chromosome haplotyping [28] was successful for 18 individuals, identifying six macro-haplogroups in the dataset: E1, J1, J2, R1 and T1. Kinship was also investigated using READ [29], but only two instances of close relationships were observed: one case a sibling relationship between an adult female (SAVBN2) and an adult male (SAVBN10) buried at Sant’Agata la Vetere and one genetic match at a tomb in Agrigento (QERBN6 and QERBN28), indicating either identical twins or one individual for whom two bones were inadvertently tested. For the following analyses, these genomic data were merged to a single file and referred to as QERBN6_28. Due to first-degree relatedness between SAVBN10 and SAVBN2, the former was selected for further analysis due to the higher number of SNPs present. Accordingly, only medieval individuals with at least 10,000 transversion SNPs, no evidence of contamination, and no first-degree relatives were included in the further analyses.

### Genetic affiliations

#### Global perspective.

**Fig 3:**
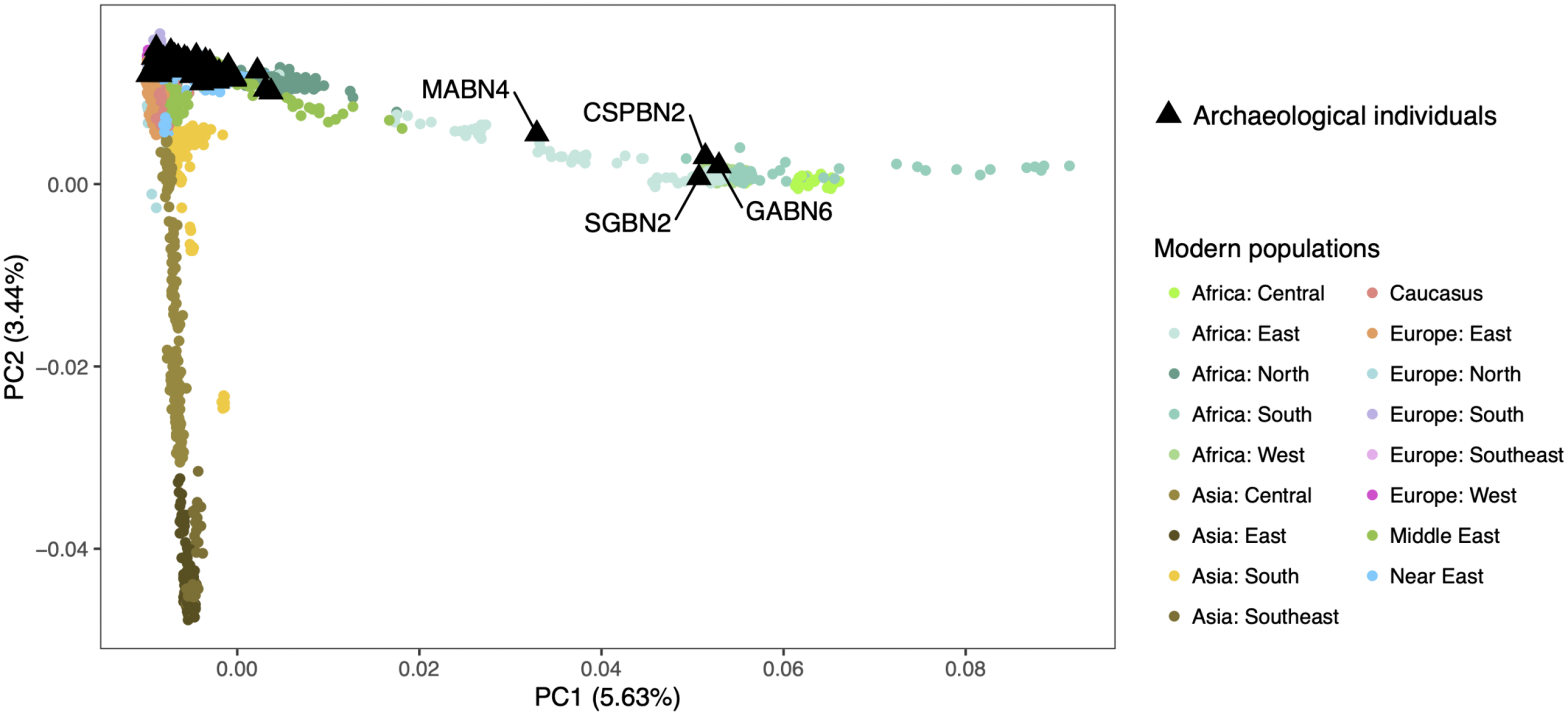
Principal component analysis of archaeological Sicilian individuals projected onto the genetic diversity of global populations. While most individuals from Sicily plot in the upper left portion of the PCA, four individuals are placed near modern populations from sub-Saharan Africa. The amount of variance explained by the first and second principal components is given in the parentheses.

DNA sequencing yielded >0.1✕ DoC on the nuclear genome for 35 individuals, thereby providing sufficient data for inclusion in admixture and continental-scale affiliation analyses [30]. Ancestry analyses were performed in conjunction with 101 published ancient genomes from Sicily: four middle Neolithic (MN) individuals, six early Bronze Age (EBA) individuals, five Middle Bronze Age (MBA) three Late Bronze Age (LBA) individuals [31], 21 Iron Age Sicani people from the sites of Polizzello and Monte Falcone, Sicily [9], 16 from Early Punic period, 9 from Late Punic period, 7 from the Punic/Roman Period, 14 from the Roman Period, 1 from Late Antiquity [10] and 15 individuals from the medieval site of Segesta [11]. After employing common methods to account for DNA damage (i.e., by evaluating exclusively transversion SNP loci in the Affymetrix Human Origins array [32]) and using sampling techniques to avoid biases introduced by different sequencing depths (i.e., by using a pseudo-haploid approach), archaeological individuals were projected onto a PCA of modern populations (S2 Table) curated in the Allen Ancient DNA Resource (AADR) database V50 [33, 34]. This analysis determined that most individuals from Sicily clustered with modern populations from Europe, the Near East and North Africa (Fig 3), while four individuals (CSPBN2, GABN6, MABN4 and SGBN2) showed affinity with African populations.

### Medieval connections with sub-Saharan Africa

**Fig 4:**
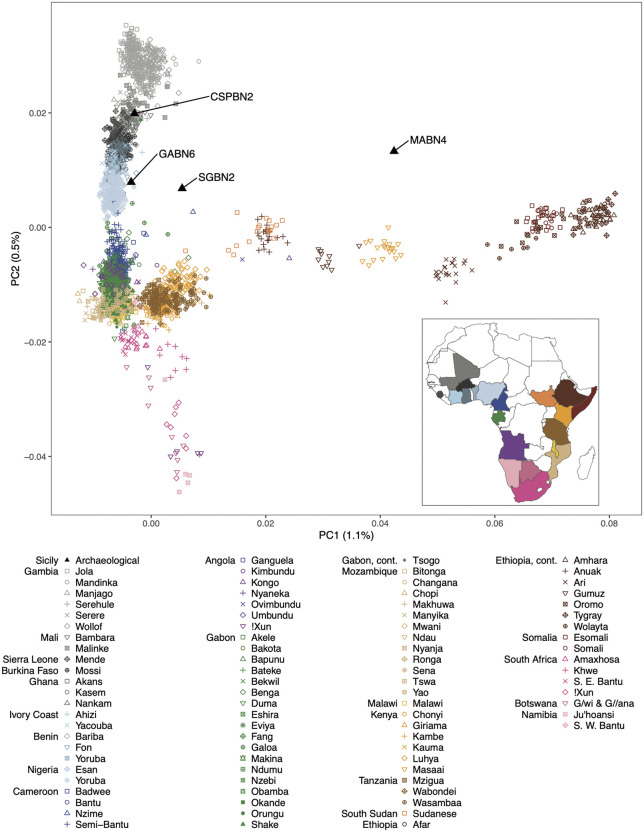
Principal component analysis of archaeological individuals projected onto the diversity of modern sub-Saharan African populations. Sandoval-Velasco et al.’s [35] database of 938 individuals from 90 populations was used for this analysis. Populations are coloured by the country of residence, as shown in the inset map. The amount of variance explained by the first and second principal components is given in the parentheses. Map created in R [12] using public domain data and ggplot2 [13], rnaturalearth [16] and rnaturalearthdata [17] packages.

We repeated the PCA with a curated African diversity panel (S3 Table) for the four individuals with putative African ancestry (Fig 4). CSPBN2, a male buried in the Islamic rite during Period 2 at Castello San Pietro, Palermo falls in PCA space within the variability of modern West African populations, plotting closest to individuals from Gambia, Mali and Sierra Leone. GABN6, a male buried in the Christian rite during Period 5 at La Gancia, Palermo also plots near groups from West Africa. MABN4, an individual buried in the Islamic tradition during Period 3 at Monte Maranfusa in western Sicily, was determined to be genetically female (XX) (but a “male” osteological assessment). This individual plots between Eastern and Western African populations. This intermediate position could reflect the absence of a closely related reference population or indicate admixed European and African ancestry. The fourth individual with African ancestry, SGBN2, is a previously published male from Segesta who was buried during Period 3 in the Islamic rite [11]. This new panel of modern African populations (S3 Table) allows us to dig deeper in the genetic affinity of this ancient individual. While the earlier analysis was limited to identifying SGBN2 as having general sub-Saharan African ancestry, this expanded reference panel confirms this observation, but also indicates that the archaeological individual plots nearest to one individual from Cameroon, three individuals from Gabon, and West African populations (e.g., Nigeria).

In addition to the nuclear DNA signals, the uniparental markers of the aforementioned individuals are common in African populations. Three individuals belong to mitochondrial macro‑haplogroup L, a group that is generally confined to sub‑Saharan Africa [36]: CSPBN2 is haplotype L3d, GABN6 is L0a and SGBN2 is L3e. The fourth individual, MABN4, differs from the others by carrying haplogroup H3, which is present in Africa, particularly in the Maghreb, but reaches even higher frequencies in Western Europe [37]. For Y-chromosome, all three males carry common African haplotypes: CSPBN2 is Y-haplogroup E1b1-P2 while GABN6 and SGBN2 are E1b1b1b1-M310.1.

Although the nuclear DNA analysis suggests that only four archaeological individuals have genetic affinities with Sub-Saharan African populations, four additional ancient individuals suggest African ancestry through their mitochondrial sequences. OBBN1, a male buried in the Islamic rite at Oratorio dei Bianchi, Palermo (Period 2) carries haplotype L3e. At the site of La Villa del Tellaro in the east of Sicily, three individuals dating to Period 2 or Period 3 belong to haplogroup L2: TLBN3 is L2e while TLBN15 and TLBN16 are L2a.

### Ancestry across the Mediterranean Basin

**Fig 5:**
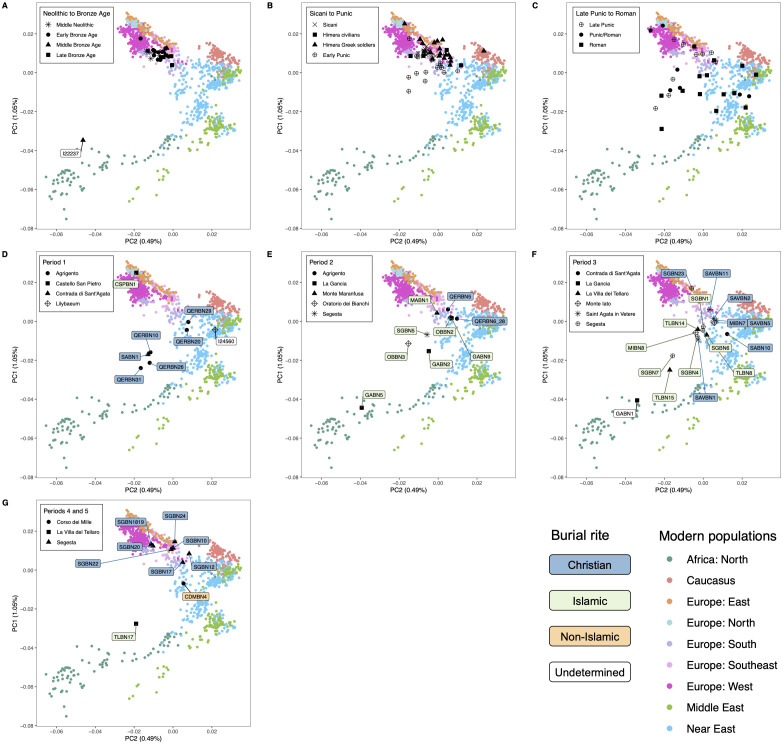
Principal component analysis of archaeological individuals from Sicily projected onto modern populations from Western Europe, Middle East and North Africa. Archaeological individuals are organised according to the chronological framework for medieval periods defined above, using the mean radiocarbon date after calibration and correction for the MRE. Individuals are labelled by site and burial rite, where “Non-Islamic” refers to the Christian or Jewish burial of individual CDMBN4. Note that PC1 is presented on the y-axis as it tracks a north-south gradient and that Period 5 is not shown as the only individual with deep sequencing data from this period (GABN6) shows a strong affinity with modern African groups. The individuals from the Neolithic, Bronze Age and Iron Age were published in previous work [9, 31], as were individuals from Segesta [11].

To further explore the genetic affinities of the medieval Sicilian individuals with putative ancestry from Eurasia, the Middle East and North Africa, an additional PCA was conducted using a modern diversity panel encompassing the region of interest (Fig 5). Individuals from local Sicilian populations dating from the Neolithic to Late Bronze Age offer a baseline for interpreting later periods, with nearly all individuals showing an affinity with modern European groups, especially populations from western, southern and southeastern Europe (Fig 5A). However, there is one outlier in this analysis, individual I22237 from Motya in western Sicily [10], who plots within the PCA-space represented by modern North African populations. This evidence for mobility or gene flow from North Africa is also observed in later periods, especially in Punic and Roman contexts (Fig 5B and Fig 5C), where many individuals plot in PCA-space between modern European and North African populations.

The earliest medieval individuals (Period 1) fall across a wide area in the PCA, but half fall into the space between modern European and North African populations (Fig 5D), similar to that observed in the Punic and Roman individuals. The other individuals from Period 1 plot within the diversity of northern and eastern Europe (CSPBN1) and the Near East (QERBN20, QERBN29 and I24560).

Periods 2 and 3 show a largely similar pattern (Figs 5E and 5F), with most individuals clustering within the spread of points representing Southeast Europe, the Near East and the same unoccupied PCA space as observed in the earlier intervals towards North African modern populations. As Periods 2 and 3 have the largest number of individuals in the dataset, one can more reliably infer that the placement of individuals across the PCA space is a sign that the genetic diversity of medieval Sicily was far more variable than seen in prehistoric Sicily (from Mesolithic to Middle Bronze Age), suggesting the sustained presence of multiple incoming populations over the centuries and a lack of rapid genetic homogenisation. This pattern is even observed within single locations, such as the wide spread of points representing individuals from La Gancia in Palermo during Period 2 and at La Villa del Tellaro in Period 3. Even though Periods 2 and 3 (Fig 5E and 5F) exhibit substantial genetic diversity, certain individuals still appear as outliers, pointing to unique origins and diverse migration patterns. In particular, two males (GABN5 and GABN1) from La Gancia in Palermo plot within the diversity of modern groups from North Africa and further carry the characteristically North African Y chromosome haplotype E1b-M81. The cemetery at La Gancia is characterised by Islamic practices, with individuals buried on their side, facing east toward Mecca, however, GABN1 was found in a supine position, and is therefore listed as having an undetermined burial rite in this study.

Period 4 and 5 (Fig 5G) are the final medieval phase studied in this article, a 100-year window including fewer individuals whom one non-Islamic from Corso dei Mille (CDMBN4, buried according to either Christian or Jewish tradition) and eight previously published ancient Christians individuals buried at Segesta [11]. Notably, the Segesta individuals [11] occupy a distinct position in PCA space, clustering with only European populations. Although it is limited and largely drawn from a single cemetery, Segesta, Period 4 and 5 (Fig 5G) lack the genetic diversity seen in earlier periods. Instead, individuals cluster with modern populations from the Near East as well as western, southern, and southeastern Europe.

From an early stage, medieval Sicily exhibits a broad genetic diversity, with most individuals aligning with a wider Mediterranean genetic profile similar to modern populations from Southeast Europe, the Near East, and towards North Africa alongside a few outliers. This pattern shows no consistent association with burial rites or time periods. Moreover, the genetic diversity observed in medieval Sicily follows similar patterns seen in Punic sites and later cemeteries.

### Identification of structured populations within non-African individuals in medieval Sicily

**Fig 6:**
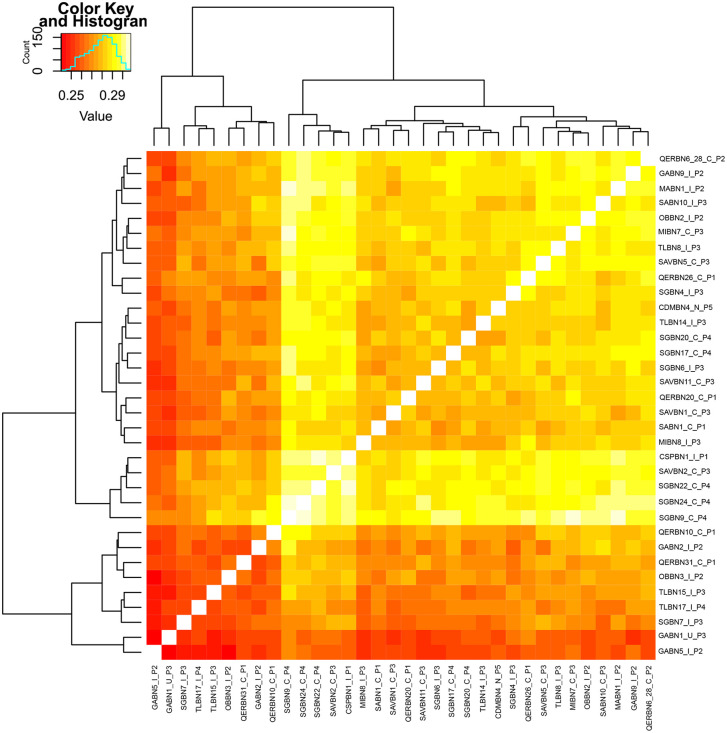
Heatmap built from F3 value; F3(Ind_X, Ind_Y; Ju_hoan_North. DG) based on 1.2 million SNPs (Allen Ancient DNA Resource, [33, 34]). Each row and column are one individual analysed. The label of the line should be read as the name of the medieval Sicilian individual (e.g., SGBN1), burial type (C for Christian, I for Islamic, U for Undetermined, N for Non-Islamic) and the period (P1 = Period 1, P2 = Period 2; P3 = Period 3; P4 = Period 4). The dendrogram was added to the left side and the top ordered based on row mean values.

We conducted an outgroup-*F*_3_ statistical analysis to formally test genetic relationships between medieval Sicilian individuals in the PCA for Western Europe, Middle East and North Africa and assess potential links between either the burial rites or time periods. The analysis was restricted to individuals with at least 30,000 transversions SNPs (n = 34 ancient individuals) and conducted using the form *F*_3_(X, Y; Ju_Huan.DG), where X and Y are medieval individuals sequenced in this study and the outgroup is a modern Juǀʼhoan individual from Namibia who is not expected to share genetic drift with any medieval individuals. The *F*_3_ values were visualised with a heatmap (Fig 6). Lighter colours indicate greater shared genetic drift between individuals (Fig 6). Most medieval individuals share some common genetic history as expected at this period in human history, reflecting shared ancestries (Western Hunter-Gatherers, Anatolian Farmers and Steppes ancestries). Nevertheless, some individuals such as GABN5 and GABN1 and five other individuals (SGBN22, SGBN24, SGBN9, CSPBN1 and SAVBN2), stand apart. Notably, the three individuals from the medieval Christian Segesta cemetery (SGBN22, SGBN24, SGBN9), along with CSPBN1 from an Islamic cemetery and SAVBN2 from a Christian cemetery, form a distinct cluster, showing closer genetic affinity to each other than to others. These results suggest that genetic relatedness among the individuals is not strongly influenced by burial rites or time period, consistent with earlier analyses.

These findings highlight that although many individuals share a common genetic history, genetic affinities in medieval Sicily transcend cultural boundaries such as burial practices and time periods, pointing to more complex and interconnected population history.

### Admixture analysis

**Fig 7:**
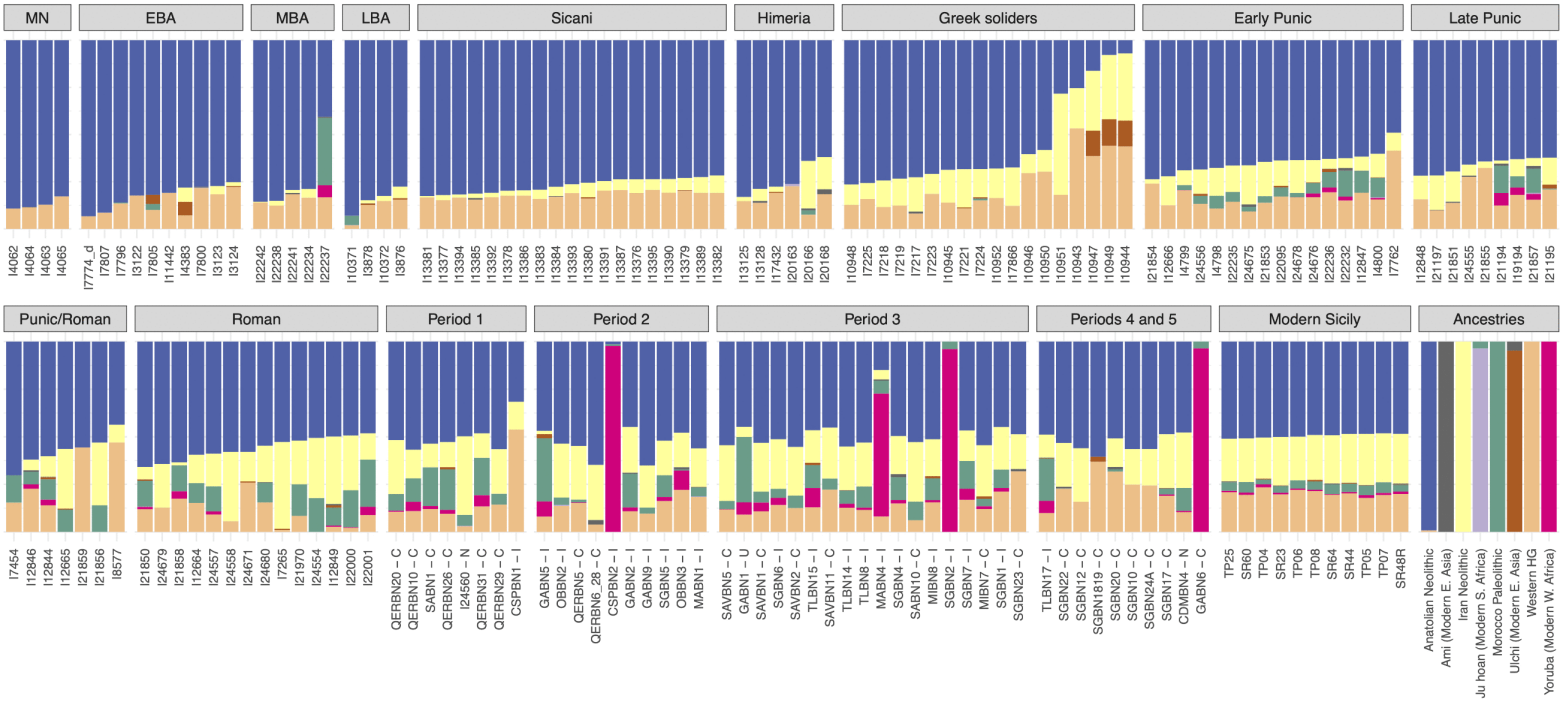
Admixture analysis of archaeological and modern individuals from Sicily. The analysis assumes *k* = 8 ancestral populations, represented by archaeological and modern ancestries at the right of the chart. The numbered periods correspond to the chronological framework for medieval Sicily described above, with other archaeological Sicilian individuals from the Middle Neolithic (MN), Early Bronze Age (EBA), Middle Bronze Age (MBA), Late Bronze Age (LBA) [31], Iron Age [9], Early Punic period, Late Punic Period, Punic/Roman Period, Roman Period, and the Late Antiquity period [10]. Medieval individuals are ordered according to the mean calibration radiocarbon age and other individuals are sorted within the respective time period by the amount of Anatolian Neolithic ancestry. The burial rite for the medieval individuals is provided after the identifier: C = Christian, I = Islamic, N = Non-Islamic (Christian or Jewish) and U = Undetermined.

As PCA can lead to artefactual associations between archaeological individuals and a reference panel [38], we conducted further investigations using unsupervised ADMIXTURE analysis in EIGENSOFT [39]. The analysis included modern and ancient populations (see details in Table S2) as well as precisely published archaeological and modern individuals from Sicily present in the Allen Ancient DNA Resource [33, 34], 11 modern Sicilians [40] and individuals who can serve as proxies for distinct ancestry components, such as Western Hunter-Gatherer (WHG), Anatolian Neolithic, and Moroccan Palaeolithic. Assuming eight ancestry components (*k* = 8), the Neolithic, Bronze Age and Iron Age Sicilian Sicani individuals demonstrate predominantly Anatolian Neolithic ancestry, with some ancestry from Western Hunter-Gatherers (WHG) and an even smaller proportion of Iran Neolithic ancestry (Fig 7).

For the earliest individuals sequenced in this article (Period 1), these three components continue to be the main ancestry sources, but variable amounts of African ancestry can be detected (Fig 7) as in the ancient Sicilian individual dating back to the Middle Bronze Age (sample ID: I22237, time: MBA) showing presence of ancient North African and Sub-Saharan ancestries and onwards in the Punic and Roman period [10]. More specifically, most of the individuals from Period 1 show the presence of North African ancestry (modelled on Moroccan Palaeolithic ancestry), and four individuals suggest a limited amount of ancestry from West Africa. In Period 2, the same ancestry components are detected across the individuals, but the ancestry proportions are more variable. For example, CSPBN1 has no African ancestry, rather exhibiting the highest levels of WHG ancestry in the dataset, GABN5 draws approximately one third of their ancestry modelled on North African ancestry, and CSPBN2 has exclusively West African ancestry. Period 3 mirrors the patterns observed in Period 2, with the same ancestry components and high levels of variability across individuals. The admixture analysis for MABN4 indicates that this individual has one of the most unique ancestry combinations in the dataset with ancestry from West Africa, Anatolian Neolithic, WHG, North Africa and Iran Neolithic. The admixture analysis for Periods 4 and 5 shows an overall decline in African ancestry, except for GABN6, who is modelled as having almost exclusively West African ancestry. The last periods also suggest lower variability in ancestry proportions and a general similarity to modern Sicilians, who despite hailing from two ends of the island, Siracusa in the southeast and Trapani in the northwest, show a remarkably consistent profile, with Anatolian Neolithic, Iran Neolithic, WHG, Morocco Palaeolithic and a small West African ancestry on average.

Overall, the unsupervised ADMIXTURE analysis identifies three key moments in the genetic history of Sicilian populations, and reveals North African and West African ancestries among medieval individuals, most notably in Agrigento (QER), even prior to the Islamic conquest.

## Discussion

This study aimed to reconstruct the genetic history of medieval Sicily in order to identify demographic shifts linked to political transitions, and more generally gain new insights into the diversity and migration patterns of medieval groups. Overall, the aDNA investigation proved largely successful, with over a third of archaeological individuals yielding sufficient nuclear genomic data to explore how regime changes shaped the island’s genetic landscape.

### Genetic landscape of Sicilian individuals across time

Using publicly available genome data for eleven modern Sicilian individuals, unsupervised admixture analyses identified five major ancestry components: Western Hunter-Gatherer (WHG), Anatolia Neolithic, Iran Neolithic, and a small proportion of North African and Sub-Saharan African ancestries (Fig 7). In a broad sense, the archaeological individuals from Sicily provide insights on when these ancestries were introduced to the island and how they changed through time (Figs 5 and 7).

The earlier periods in this dataset (MN, EBA, MBA, and LBA) are dominated by the Anatolian Neolithic component with a smaller contribution of WHG ancestry. As anticipated by Moots et al.’s model of the genetic history of the central Mediterranean [41], the Iran Neolithic ancestry component was introduced to Sicily by the Iron Age. The Sicani Iron Age individuals consistently have a modest amount of this ancestry component, but neither they nor earlier individuals are found to have predominately Iran Neolithic ancestry. The Iran Neolithic ancestry component is found to have an early peak within some Greek soldiers, but otherwise increases through the Roman period, and ultimately stabilises by the Middle Ages. This observation is consistent with Moot et al.’s [40] interpretation of a shift toward eastern Mediterranean ancestries in the Roman Empire through gene flow.

The cumulative impact of eastern ancestries introduced during the Neolithic, Bronze Age, Iron Age to the Roman Period creates a complex genetic background that can make it more difficult to disentangle the later contributions from the Near and Middle East in Sicily during the Middle Ages.

Interestingly, the North African and Sub-Saharan African ancestries observed in modern Sicily are similar to the ratios observed in many archaeological individuals following the Iron Age (e.g., I24678 from the Early Punic, QERBN5 from Period 2, and SGBN6 from Period 3). The presence of North African ancestry in Sicily may reflect migrations associated with Phoenician-Punic [10, 31, 42] and Roman [43] expansions, although Middle Bronze Age individual I22237 from Motya in western Sicily [10] shows the presence of North African ancestry (Fig 7) suggesting individuals may have moved long distances in earlier periods. The presence of North African ancestry raises the question of whether it reflects a broader Sicilian genetic landscape or if it is specific to certain regions of the island.

Sub-Saharan African ancestry starts to be detected in Sicily during the early Punic Period, but it is only in the early Islamic cemeteries that individuals exhibit exclusively Sub-Saharan African ancestry. The timing of the movement of people from Sub-Saharan Africa to Sicily is difficult to infer, although within this dataset the first individual with exclusively Sub-Saharan African ancestry, CSPBN2, dates to medieval Period 2, in one of the earliest Islamic cemeteries in Palermo. Nonetheless, these ancient DNA results are largely compatible with Hellenthal et al.’s [44] admixture analysis which supports multiple pulses of African ancestry into Sicily from the second century CE to the late medieval period. Interestingly, a similar pattern has been observed in Spain [45] and southern Portugal [46], where Sub-Saharan African ancestry is only detected only after the Islamic conquest.

The PCA (Fig 5) and unsupervised admixture (Fig 7) analysis highlight also the dynamic period spanning the Greek, Punic, and Roman civilisations to the Middle Ages itself leaving an indelible mark across the Mediterranean. Thus, additional fine scale palaeogenomic studies are needed to better understand exactly how the genetic landscape of the interconnected Mediterranean world was established at a fine scale. It is also worth noting that the reference panel for modern Sicilians has an obvious shortcoming with sampling restricted to just two cities, and more expansive testing across the island could shed light on heterogeneity between communities or ancestry components that mirror other archaeological individuals.

### Burial traditions and lineages

By integrating genome-wide genetic data, burial contexts, and uniparental markers, one can explore beyond ancestry components to infer the longevity of cultural traditions and impacts of regime change.

First, our results show that burial practices generally do not align to specific genetic groups. For example, Byzantine Christian individuals from Agrigento (Period 1, Fig 5D) occupy the same PCA space as earlier Punic and Roman individuals, as well as later individuals buried with Islamic rites in Period 3. Additionally, some Period 1 and 2 individuals with Islamic rites (e.g., CSPBN1 and MABN1 in Fig 5D and 5E) cluster with modern European populations. Finally, in Period 3 (Fig 5F), a Christian individual (SAVBN1) plots alongside medieval Islamic individuals. As we previously reported [11], it is only at Segesta during Periods 3 and 4 (Figs 5F and 5G) where burial practices cluster by ancestry. Nevertheless, the overall limited correspondence between ancestry and burial rites highlights the need for further palaeogenetic investigations of medieval contexts to better understand the dynamics of faith groups.

For uniparental lineages, this study again does not find consistent patterns between burial rites and genetic markers for most individuals. The noteworthy exception is that individuals with haplotypes associated with African populations, mtDNA haplogroups L3e/d and L2 and Y-chromosome haplotypes E1b-P2 and E1b-M81, were predominantly buried following Islamic rites. The presence of Y-chromosome haplotype E1b-M81 in Islamic cemeteries at La Gancia in Palermo (GABN1 and GABN5) and Segesta (SGBN1 and SGBN3) is particularly intriguing, as Gleize et al. [47] and Olalde et al. [45] have proposed this haplotype tracks the Islamic expansion. However, for the African mtDNA haplogroups, a recent synthesis of mtDNA data by Tommasi et al. [48] demonstrates the presence of characteristically African haplotypes in the MBA [49] and Roman [50] periods.

### Genetic traces of distant origins

The genetic outliers in our dataset underscore Sicily’s position as a hub of medieval migration, connecting people from across Europe, the Near East and Africa.

In two of the earliest Islamic cemeteries in this study, La Gancia and Castello San Pietro, five individuals with distinct ancestries provide invaluable narratives on the great distances they or their immediate ancestors must have travelled. At La Gancia in Palermo, two of the three medieval individuals (GABN5, Period 2 and GABN1, Period 3) closely match within the genetic diversity of North African populations (Fig 5E and 5F, Fig 7). The other individual from La Gancia, GABN6 from Period 5, draws nearly 100% of their ancestry from Sub-Saharan Africa. This pattern is similarly observed at the Castello San Pietro Islamic cemetery in Palermo, where CSPBN2 (Period 2) has nearly 100% Sub-Saharan African ancestry. Another individual buried with Islamic rites at Castello San Pietro, CSPBN1, presents a dramatically different perspective on the diverse genetic origins at the site. In contrast to the African links described above, CSPBN1 is remarkable for completely lacking North African and Sub-Saharan African ancestry components, and instead has a distinct Northern/Eastern European ancestry (Fig 5D) with typical European Y-chromosome haplogroup R1a-M17. Both CSPBN1 and CSPBN2 have no signs of recent admixture (Fig 7), and instead likely represent individuals with direct links to Northern/Eastern Europe and Sub-Sahara African, respectively. Di Salvo [51] has argued that the Castello San Pietro cemetery may represent a family group with eunuchs, which could explain this unusual genetic history; however, with only two individuals, it is difficult to assess this hypothesis.

While the admixture analyses indicate that modern Sicilians can trace their genetic origins to a series of historical events, only one medieval individual provides clear evidence for procreation between groups. Specifically, MABN4, a female from Monte Maranfusa (Period 3), shows an ancestry profile compatible with them having similar amounts of both European and African ancestries. While intermarriage is documented in historic sources [e.g. 3, 6, 52], this study provides only limited evidence for gene flow between groups. However, as previously discussed, the ancestry components of different groups are overlapping, thereby making it challenging to identify admixture between more closely related groups. Nonetheless, the presence of individuals with unique ancestry profiles at La Gancia, Castello San Pietro, Monte Maranfusa and Segesta (SGBN2) [11] provides genetic evidence for long distance migration following the Islamic conquest.

Even though the diverse ancestral origins lead to an inference of long-distance migration, the genetic analyses are unable to resolve whether individuals like CSPBN2 travelled thousands of kilometres from West Africa to Palermo or if their parents or grandparents made such a journey. Expanded study of human remains with geographically informative stable isotopes could help resolve the degree to which individuals moved great distances around the Mediterranean in these periods. By evaluating stable isotopes of individuals, a better understanding of migration and gene flow in the Middle Ages could be achieved.

A final and sensitive consideration of this study is that we may never fully appreciate the hardships these individuals may have endured during their lives. It is well established that Sub-Saharan Africans were enslaved and forcibly transported by the Garamantes to the Roman world already by the first century CE [53], and both African and European individuals were enslaved during Islamic rule [54, 55]. The historical context of slavery in the Middle Ages provides some useful perspectives, as among Islamic literature scholars, there is a discussion on the term “Ṣaqāliba”, which can be interpreted as referring to enslaved Slavic individuals or a broader group of enslaved people, including eunuchs and other enslaved Europeans [56, 57]. On the other hand, some medieval individuals moved long distances through their chosen occupation or otherwise on their own volition, such as through Berbers in Islamic armies [1, 5], merchants and North African individuals fleeing famine and war [1]. Therefore, determining the social status of medieval individuals, whether they were free or enslaved, is impossible with the available data. For example, the burial rites of individuals with distinct ancestries do not differ from others with their respective cemeteries. Given the uncertainties in the terminology and the non-distinct burials of the genetic outliers, one cannot conclusively infer the societal status of these individuals or how they may have come to live in Sicily. Still, expanded DNA sampling and integration of stable isotope analysis could help refine the understanding of the timing and scale of long distant movement to the Mediterranean region.

### Concluding remarks

Overall, our study has provided new insights into the medieval Sicilian genetic landscape, showing above all that Sicily was an important melting pot during the Middle Ages, continuing a tradition of movement seen in Greek, Punic and Roman periods. One of the most notable findings is the presence of North African and Sub-Saharan ancestries in southern Sicily prior to the Islamic conquest, although African ancestries were observed in one MBA individual [10]. Interestingly, while these African ancestries were present in Sicily before the Islamic period, individuals buried according to Islamic rites frequently carried African mitochondrial DNA or African Y-chromosome haplogroups. These data suggest that the Islamic conquest did not result in a large-scale genetic replacement of the contemporaneous Sicilian population. However, the relative scarcity of Christian individuals from some periods in this dataset limits the strength of this conclusion. To better understand population dynamics, future research should include a broader sampling of Christian burials from across the island, combined with isotopic analyses (oxygen and strontium) to investigate mobility and identify potential first-generation migrants, especially among individuals presenting a distinct genetic pattern. Expanded genetic testing of archaeological individuals from Sicily and across the Mediterranean basin could also provide finer resolution on the ancestral origins of individuals and the detection of kinship across multi-faith communities. At the site of Segesta [11], there is evidence of genetic structuring by burial rites during the late medieval period, underscoring the need for further focused studies. Additionally, comparing medieval Sicilian genomes with more contemporary populations in the wider region will enhance our understanding of the genetic impacts of successive conquests in the broader Mediterranean area.

## Materials and methods

Additional information regarding the ethical, cultural, and scientific considerations specific to inclusivity in global research is included in the Supporting Information (S2 File).

All necessary permits were obtained for the described study, which complied with all relevant regulations. Permission for destructive sampling were granted by the Soprintendenza di Enna, Museo Archeologico Antonino Salinas Palermo, Museo Regionale Paolo Orsi Siracusa, Parco Archeologico della Valle dei Templi Agrigento, Soprintendenza di Palermo, Soprintendenza di Trapani and Università di Catania. Further details on the authorisations, permit numbers and repositories are provided in S1 Table.

The ancient DNA and radiocarbon methodologies followed the approaches employed by Monnereau et al. [11] (see S1 File for full details). Archaeological remains were tested from 18 sample collection locations, as described in S1 File. Radiocarbon dating was undertaken with accelerator mass spectrometry (AMS) at SUERC, the University of Glasgow (UK). Radiocarbon measurements were calibrated with IntCal20 atmospheric curve [58] using OxCal version 4.4, including corrections for marine reservoir effects and implementation of Bayesian inference [19, 59]. Palaeogenetic analyses were performed at a dedicated ancient DNA laboratory at the BioArCh Facility, University of York, UK (for further details on the protocols used please see S1 File). DNA recovered from archaeological bone was converted to high throughput DNA sequencing libraries and sequenced on Illumina platforms. For libraries with low endogenous DNA content, in-solution enrichment of mitochondrial DNA was performed. Bioinformatic analyses followed the methods described by Monnereau et al. [11], with an additional investigation of sub-Saharan ancestry using a curated database of modern African populations [35]. Ancient individuals that exhibited the expected patterns of DNA degradation and demonstrated no contamination of the mitochondrial genome and/or X chromosome were included in the analyses.

## Supporting information

S1 File Supporting text. Detailed description of archaeological context, radiocarbon methodologies, ancient DNA laboratory methodologies, bioinformatic steps and expanded kinship analysis.
(PDF)

S2 File Checklist. Information regarding the ethical, cultural and scientific considerations specific to inclusivity in global research.
(PDF)

S1 Table Archaeological and genetic individual summaries. Summary of the archaeological context, genetic and datation analyses of the new individuals included in the study.
(XLSX)

S2 Table Reference populations. List of modern and ancient populations (at least 10,000 transversion SNPs) from AADR database V50 that were used in this study.
(XLSX)

S3 Table African reference panel. List of modern African populations from Sandoval-Velasco et al. (2023) that were used in this study.
(XLSX)
