# Supplementary material for: Genetic histories of individuals from multi-faith medieval Sicily
Source: PLoS One. 2026 Jun 24;21(6):e0350298. doi: 10.1371/journal.pone.0350298 (PMC13293407; doi:10.1371/journal.pone.0350298)
Supplement: S1 File — Detailed description of archaeological context, radiocarbon methodologies, ancient DNA laboratory methodologies, bioinformatic steps and expanded kinship analysis. (PDF) [file pone.0350298.s001.pdf]

S1 File. Supporting text for "Genetic histories of individuals from multi-faith medieval Sicily"

Aurore Monnereau[1*], Paola Orecchioni[2], Derek Hamilton[3], Eleanor Joan Green[1], Ian Noble[1], Richard Hagan[1], Marcela Sandoval-Velasco[4,5], Alessandra Molinari[2], Martin Carver[1], Camilla F. Speller[1,6], Nathan Wales[1 *]

[1]BioArCh, Department of Archaeology, University of York, York, United Kingdom

[2]Dipartimento di Storia, Patrimonio Culturale, Formazione e Società, Università degli Studi di Roma Tor Vergata, Rome, Italy

[3]SUERC, University of Glasgow, Glasgow, United Kingdom

[4]Center for Genomic Sciences, National Autonomous University of Mexico, Cuernavaca, Mexico

[5]The GLOBE Institute, Faculty of Health and Medical Sciences, University of Copenhagen, Copenhagen, Denmark

[6]Department of Anthropology, University of British Columbia, Vancouver, Canada

* Corresponding authors

E-mail: aurore.monnereau@palaeome.org (AM), nathan.wales@york.ac.uk (NW)

This PDF file includes:

Supporting text ; Tables A to E; SI References

Other supporting materials for this manuscript include the following:

S1 Table; S2 Table; S3 Table

Archaeological context

This component of the project was led by Martin Carver, Alessandra Molinari, Paola Orecchioni and Aurore Monnereau.

The human remains analysed in this study originate from multiple archaeological sites in Sicily, provided for scientific analysis by collaborating researchers and excavators. The collaborators and excavators who granted access to the human remains for this European project are acknowledged in the acknowledgements section of the main article. All information related to the archaeological human remains can be found in the S1 file, S1 Table and in NCBI bank project PRJNA1400023. The osseous remains, DNA extracts and libraries are stored at BioArCh, University of York (UK).

In total, 111 individuals buried in the Christian and Islamic traditions were selected for analysis (S1 Table; Figs 1 and 2). Tombs identified as belonging to an Islamic rite are characterised by positioning the body of the dead on the right side and with the head facing Mecca, while Christian tombs are characterised by a supine position of the body.

Short descriptions of the archaeological sites are presented below, ordered as in Fig 1, which largely follows a west-to-east direction.

San Miceli (SM) (Christian cemetery) (Fig 1. N° 1)

San Miceli was a significant rural settlement that included a residential area, and a place of worship with a baptistery, and a cemetery [1]. The site began developing at least as early as the 3rd century CE with the

construction of a rural villa. In the second half/end of the 4th century, a small baptismal and funerary church transformed the rural landscape. After destruction in the second half/end of the 5th century, the settlement was rebuilt in the 6th century and remained occupied until its final abandonment in the late 7th century. Excavations by A. Salinas in 1893 uncovered the church and sixty-five tombs, while more recent work by Elisabeth Lesnes and Randall Younker since 2014 revealed additional tombs and burials in stone and earth pits. Tombs were found within the aisles of Basilica 1 and 2 and outside the west apse and east wall of the nave. Tomb 1 in the narthex contains a man and a woman, the latter buried with silver earrings, a jug, a ring, and a coin (Aes 3 of Constantius II or Flavius Claudius Julianus, dating to 355-361 AD). Tomb 2 contained two men and a necklace fragment, while Tomb 4 held a woman with more elaborate earrings, a jug, and half a ring. Two shallow graves in the north aisle contained children and the disturbed remains of an adult woman, dug through the mosaic floor of Basilica 2. They are interpreted as hasty burials made after the 7th-century episode of destruction. Two ancient individuals were analysed for aDNA, SMBN1 (775-1005 CE with marine correction) and SMBN4 (S1 Table)

Monte Maranfusa (MA) (Islamic cemetery) (Fig 1. N° 2)

Monte Maranfusa is a hill located in western Sicily, reaching an elevation of 487 m asl. The archaeological site was investigated between 1986 and 2008 in seven interventions (Campi A-H). Burials consistent with Islamic funerary practices were found in three interventions (Campi A, G and H) [2,3]. For ancient DNA (aDNA) analysis, skeletal remains were obtained from Campo A, where 17 individuals were recovered from 21 graves cut into or adjacent to the walls of an earlier archaic settlement, abandoned approximately 1,500 years prior. The individual skeletons were found in graves of curvilinear shape, sometimes lined with small stones, most covered by horizontal or tilted stone slabs. Individuals were interred on their right side, with the head oriented westward and the face turned south. In Campo G,

the 16 burials were oriented with the head to the southwest and the face to the southeast. The variation in Campo A may have been influenced by the alignment of the earlier walls [4,5]. Four individuals were selected for analysis [5]; they were radiocarbon dated to the 10th–13th centuries CE.

Monte Iato (MI) (Islamic and Christian cemetery) (Fig 1. N° 3)

Monte Iato is represented by distinct Islamic and Christian cemeteries. In contrast to Segesta—where the Islamic cemetery predates the Christian cemetery by at least a century and ends by the mid-13th century—radiocarbon dates from Monte Iato suggest that the Islamic and Christian cemeteries were broadly contemporaneous, spanning from the mid-11th to the mid-13th century. Although the precise end dates are uncertain, documentary sources indicate the settlement was likely abandoned in the mid-13th century [6]. As at Segesta, the Islamic cemetery (Cemetery 1) was located along the outer rim of the Greek theatre, while the Christian cemetery (Cemetery 2) was situated in the former Agora, adjacent to medieval dwellings [6]. In Cemetery 1, individuals were interred on their right side with the face turned southeast, typically in stone-lined graves. In Cemetery 2, individuals were buried in a supine position, also in stone-lined graves, with the head oriented east or southeast. Remains from 12 individuals (four male, three female, and five of undetermined sex) were recovered from Cemetery 1, and 22 individuals (four male, nine female, one child, and eight undetermined) from Cemetery 2 [5; Mölk, pers. comm.,7]. Seven individuals were radiocarbon dated from 11th to the 13th CE) and analysed in this study—four from Christian cemetery and three from Islamic cemetery.

Monte Iato Castellazzo (MIC) (Islamic burial practice) (Fig 1. N° 4)

At Monte Iato Castellazzo (MIC), a body (MIC_BN1) was laid face down with the face turned towards the south, close to him was a broken iron arrowhead. The body position suggested an Islamic burial rite [8]**.** He was dated from the 11th to the 13th CE.

Contrada di Sant'Agata, Piana dei Albanesi (SA) (Christian cemetery) (Fig 1. N° 5)

Contrada di Sant'Agata, Piana dei Albanesi or in short, Contrada Sant'Agata is located 30 km south-west of Palermo. Following activities of looters, the Soprintendenza initiated formal investigations in 1988–1989. Surface survey revealed evidence of adjacent settlements spanning from the Hellenistic to the late medieval period, covering an area of approximately four hectares southeast of the cemetery [9]. The cemetery comprises more than one hundred stone-lined tombs, originally marked with stone heaps—a mortuary tradition reminiscent of North African practices. The burials were arranged in rows, interpreted as representing family groups [10]. A spatial distinction in burial orientation was observed: graves in the northern section were aligned north–south, whereas those in the southern section were oriented northeast [10]. Nine individuals were selected for biomolecular analysis. Radiocarbon dates range from 5th to the 7th century CE, with the exception of one male individual (BN10/T183) dated to 1050–1260 cal CE, and one female individual (BN3/T182/3) dated to 270–520 cal CE. In total, nine individuals were analysed in this study, including three from Tomb 182 and two from Tomb 77.

Palermo

Palermo, the capital of Sicily, is situated at the north-west coast of the island. Palermo fell to Muslim troops in 831 CE. Under the Shi'ite Fatimid rules, Palermo (or Balarm) saw some urban change, including the foundation of a fortified citadel, the Khalisa (937-938 CE) outside the city walls. Palermo under

Islamic rule was divided into different parts. The centre of the city was surrounded by nine gates and four suburban areas [11,12]. Medieval Christian burials have been found inside the fortified city, and to north and south of it. The cemetery at Via Guardione, outside the city walls to the northeast and excavated under modern conditions, contained Christian burials radiocarbon-dated in the range 7th to 12th centuries CE. Islamic cemeteries have been found in three groups, all outside the city walls: to the north-east (Castello San Pietro), to the east (La Gancia, Oratorio dei Bianchi) and to the south (Corso dei Mille). A second cemetery at Corso dei Mille contained non-Islamic (Christian or Jewish) burials. Bayesian modelling of the radiocarbon dates has shown that Islamic burial progressed between the 9th and 13th centuries from the north-eastern, to the eastern and then the southern group. These cemeteries are described in more detail below.

Castello San Pietro (CSP) (Islamic cemetery) (Fig 1. N° 6)

Castello San Pietro, located in Palermo, was excavated in two areas known as Saggio A and B. In Saggio B, walls defined rooms designated as Vani I–IV, and 12 earth-cut burials were uncovered containing the remains of 13 individuals. The funerary practices observed correspond to Islamic traditions, with most individuals positioned on their right side facing southeast, except for the children, who were laid in a supine position. The excavators identified nine males, two females, and two children. Based on cranial morphology, Arcifa and Bagnera [13] and Di Salvo [5] suggested a North African origin for this population. Three individuals were selected for radiocarbon dating, with results ranging from 7th to 11th centuries CE. However, dates earlier than 831 CE —the date of documented Muslim entry into Palermo— were considered unlikely. Three individuals (BN1/T4, BN2/T6, BN4/T10; two males and one female) were submitted for ancient DNA analysis. Radiocarbon dating places these remains within the 7th to 11th centuries CE, consistent with the timeline of the conquest of Palermo in 831 CE.

La Gancia (GA) (Islamic cemetery, Christian cemetery and one individual, GABN1 with an undetermined burial practice) (Fig 1. N° 7)

La Gancia — the abbreviated name used in this study — refers to an Islamic cemetery located around the Church of Santa Maria degli Angeli della Gancia. Seven archaeological interventions were made during the restoration of the church, yielding the remains of nine individuals [5,14]. In the Saggio G, two burials were uncovered: adhering to Islamic funerary practices, BN5 (G338) was radiocarbon dated to the 7th–10th centuries CE; stratified above this burial, another individual (BN1/G303) was recorded: a male in a supine position with the head, to judge from the skeleton, towards the north-west, and dated to the 10th-12th centuries CE. The burial rite of this individual is uncertain. In Saggio C, six burials were identified, of which five individuals were analysed (C397-401, C402, C381/IndA, C381/IndB and C474) dating from 8th-12th centuries CE. Burial C402, an infant was in a supine position, although children buried in a supine position encountered in Islamic cemeteries are not thought to be Christian. All these burials, with the possible exception of G303, were consistent with the Islamic rite, and their dates consistent with the documented dates of Islamic governance. The ninth burial, BN6 (H227), buried in the Christian rite, was dated between the 15th and 18th century CE.

Oratorio dei Bianchi (OB) (Islamic cemetery) (Fig 1. N° 8)

The Oratorio dei Bianchi, constructed in 1542 within the Church of the Madonna della Vittoria (built in 1489), contained a Muslim cemetery uncovered during excavations in 1997. At least 19 earth-cut graves were identified, some covered with limestone slabs and, in five cases, with slabs tipped obliquely within the grave [14]. The individuals were interred according to Islamic funerary practice, lying on their right side with the face oriented southeast. The skeletal remains were in poor condition; among those recovered, only three adult males, two females, and two neonates could be identified [5]. Three

individuals (BN1, BN2 and BN3) were submitted for biomolecular analysis. The radiocarbon dates range from 8th to 11th centuries CE.

Palazzo Abatellis (PA) (Islamic cemetery) (Fig 1. N° 9)

Located near La Gancia and the Oratorio dei Bianchi, 15 Islamic burials were identified in the courtyard of Palazzo Abatellis. The individuals were interred according to Islamic rites, with their heads oriented southwest and faces turned southeast [5,14]. One individual's remains were submitted for analysis; however, insufficient material was available for radiocarbon dating or ancient DNA analysis.

via Guardione (VG) (Christian cemetery) (Fig 1. N° 10)

In 2018 during excavations for sewage works, a cemetery about 120 m^2 in extent was uncovered to the north of the Piazza XIII Vittime, near the junction between via F. Guardione and via F. Crispi. The graves were cut into limestone bedrock arranged in orderly rows orientated E-W and covered with stone slabs. Around 62 graves were defined, of which 40 contained a single body, twelve had two bodies and twelve had multiple occupants, amounting to about 100 skeletons in all. Three individuals (Burials 50, 54 and 32) were submitted for biomolecular analysis dating from 7th to the 13th centuries CE.

Corso dei Mille (CDM) (Islamic and Christian or Jewish cemetery) (Fig 1. N° 11)

Approximately 30 burials were uncovered during rescue excavations along Corso dei Mille associated with tramway construction. Of these, 22 were supine with heads oriented southwest, most contained within wooden coffins, consistent with Latin Christian or Jewish burial practices [15]. An additional four

burials were positioned on their right side with heads facing west to southwest and faces oriented south, interpreted as Islamic interments. One of the supine burials contained an Egyptian perfume bottle dated to the 12th–13th centuries CE [16]. Remains from nine individuals were submitted for ancient DNA analysis, of which eight from supine burials and one from a right-side burial. The eight supine burials dated between the 11th and 15th centuries CE. The only burial following Islamic burial rite (BN4/Civ 120/Ind 3) was dated 1250-1405 cal CE. The single Muslim burial was dated from 11th to the 13th centuries CE. Nevertheless, only four yielded sufficient endogenous DNA for further analysis (BN1, BN4, BN7, and BN16).

Castronovo di Sicilia (Christian and Islamic burial practices) (Fig 1. N° 12 and N°13)

Two burial sites have been investigated at Castronovo di Sicilia in the central-west part of Sicily: Colle di San Vitale (SV), Casale San Pietro (CLESP). At Colle di San Vitale, there was a single male skeleton (SVBN1) whose head was oriented to the west, and radiocarbon dated 1300-1440 CE. Casale San Pietro (CLESP) is a village beside the river Platani where it is crossed by the road linking Palermo and Agrigento. The tombs of two children were excavated in the field adjacent to the Casale [17]. An adult skeleton (CLESPBN1) was exposed in the former church at the Casale was radiocarbon-dated 1690-1935 CE and did not have any DNA (i.e., to the modern period).

Agrigento (QER) (Christian cemetery) (Fig 1. N° 14)

Agrigento is situated on the south-central coast of the island and has a long history of burials dating back to the Archaic period. Approximately 50 early medieval burials have been identified within the rooms of abandoned houses in the former Greek and Roman town known as the Quartiere Ellenistico-Romano

(QER), located between the modern town of Agrigento and the Valley of the Temples. The observed funerary practice includes large, free-standing tombs constructed with mortared stone walls, aligned with the orientation of the houses and street grid, which generally runs north–south and east–west. The tombs contained men, women, and children, likely related by kinship [18–20]. Three burial groups were investigated, located in Insulae I, II, and III. Remains from 25 individuals were analysed through ancient DNA analysis and recovered from 9 tombs—two in Insula I (House IC), five in Insula II (Houses C and D), and two in Insula III. Of these, 18 individuals were radiocarbon dated. According to Bayesian modelling conducted by SUERC, the use of the QER cemetery began between 235-405 CE and ended between 775-1000 CE (95% probability). The sequence of use followed the order: Insula II, Insula I, and finally Insula III. In this paper, descriptions for each Insula are presented in the chronological order suggested by the radiocarbon dates.

Insula II, used between the 3rd and 6th centuries CE, contained five tombs distributed across two houses: Casa II D and Casa II C. The tombs were found in four rooms—Casa II D, room a; and Casa II C, rooms h, a1, and n1. Tomb 1, Insula II (Tomb II/1) in house/Casa II D, room a, probably a vestibule, contained two burials: first individual 6L, followed by 5L (BN17; S1 Table to associate with sample ID for all samples). Tomb II/2 situated in house/Casa II C, room h contained six individuals deposited in three phases. The first phase is characterised by the deposition of a male individual (13L, BN10) with an injury on the skull, then an infant (11L, BN12) present at the base of the tomb. The other phase contains some disturbed bones. Remains of two individuals were submitted for aDNA analysis: one bone of a child (9L, BN24), and the other of a possible adult (9L, BN11). A female (10L, BN9) laid over them accompanied by a hairpin, gilt bronze earring and a brooch. Tomb II/3 is also situated in the same house and room. The first deposition is made up of disarticulated bones (31L, BN20) followed by an articulated skeleton of a male (28L, BN19). No remains were submitted for aDNA analysis from Tomb II/4. In Tomb 5/II (Casa II C, room a1), two superimposed children (48L, BN15; 49L, BN16) were uncovered. In Casa II C, room

n1, Tomb II/6, three children were recovered. Two children were close in age (6N,BN22; 8N, BN23; around 4 years old) with a third child (7N, BN21) buried above them [19].

Insula I burials were dated between 5th–8th centuries CE, the inhumed individuals were also aligned west-to-east in room r of Casa IC. Tomb I/1 contained six individuals: two females (109a, BN26 and 106a, BN2), one male (110a, BN1), one individual of unassigned sex, and two children (104a, BN30 and 111a, BN31). Tomb I/8 contained one male (6c, BN27).

The last insula, Insula III was dated between the 7th-11th centuries CE [21]. Tomb III/8 (4c, BN7) was in an Ambitus containing a male aged between 25-35 years. Tomb III/3 contained the remains of seven individuals, deposited in the following order based on radiocarbon dating: two females (5a, BN29 and 16a, BN5), followed by another female (17a, BN4), a male (18a), and finally two young adult females (8a, BN6 and 9a, BN28), buried together and both estimated to be around twenty years old.

Although the multiple use of the tombs usually prevented the direct association of individuals with the few grave goods, the use of aDNA enabled the detection of changes in a Byzantine cemetery over 500 years using well stratified radiocarbon dating. In total, 25 ancient individuals were analysed.

Enna (ENN) (Christiana and Islamic cemetery) (Fig 1. N° 15)

During recent water pipe installations in the Mulino a Vento district of Enna, human burials were uncovered in three areas: two on Via Ragusa (SAS I and II) and a third in Piazza Santa Sofia (SAS III). Though close to the surface and partially damaged by roadworks, the burials were archaeologically investigated under Dr. Rossella Nicoletti [22]. Evidence suggests these graves were part of a larger cemetery, with ten individuals buried in the Islamic rite—on their right side, heads to the southwest, and

faces south/southeast, with no grave goods. Four others were buried supine and are proposed to be Christian. In SAS I, the earliest burial (Burial 2) followed Islamic burial practice, while later burials (1 (BN1), 3, and 4) were Christian and dated at least a century later. SAS II revealed six burials, five of which were following Islamic funerary practice, including possible lahd burial, while one later Christian burial (Burial 6, BN6) lay above an earlier grave. Precisely, the head of Burial 5 was missing and only the head survived of Burial 7 (BN7), all five were buried in the Muslim rite, laid on the right side with the head towards the South-West and the face towards the South-East. Burial 9 (BN9) had been reburied with the head placed in the Muslim manner in a trapezoidal grave. SAS III, on the edge of the Piazza Santa Sofia, contained four Islamic burials, including BN12 and another burial 14 (BN14) likely lahd burial. Radiocarbon dating of nine individuals placed the Islamic burials in the 10th-12th centuries CE. For the Christian ones, the range is between the 15th and 17th centuries CE. This indicates a Christian cemetery succeeded an earlier Muslim one. Attempts to extract ancient DNA from the remains were unsuccessful.

Sant'Agata La Vetere (SAV) (Christian cemetery) (Fig 1. N° 16)

The archaeological site of Sant'Agata La Vetere is located in Catania. In this site, two medieval Christian cemeteries were located, termed Cemetery 1 and 2. Cemetery 1 consisted of 11 stone-lined and capped tombs [23]. Cemetery 2 was situated adjacent. Here excavators encountered human bone in seven rectangular tombs with stone linings bonded with a coarse mortar containing fragments of bones and ceramics. There was also an ossuary or mass grave (Tanasi pers. comm.).

Twenty-six human remains were received from Cemetery 1 in two batches, 11 from batch 1 and 15 from batch 2. Nine human remains from Batch 1 were analysed by aDNA. Six were successfully radiocarbon dated from 10th-13th centuries CE. Ancient individuals BN2 (dated 1035–1220) and BN10 (dated

1035–1230) were shown to be related in the first degree. All the human remains confirmed the date of cemetery 1.

Villa del Tellaro (TL) (Islamic cemetery (Fig 1. N° 17) and Roman cemetery (Fig 1. N° 18))

La Villa del Tellaro, located in the south-eastern part of the island, revealed 30 burials situated beside the road leading to a nearby former Roman villa. The graves were spatially organised into four main groups, with four outliers. The largest cluster (Group 1) was centred around a pool, a basin, and a water channel. Radiocarbon dating of 20 individuals showed that two outliers (T13, BN19 and T13bis, BN20) dated to the Roman period (110 cal BCE–115 cal CE). The remaining two outliers (T12, BN8 and T24), as well as all individuals within the four burial groups, were identified as Muslim burials, dated broadly between the 10th and 13th centuries CE. Bayesian modelling indicates the cemetery was in use from 895-1015 cal CE and continued until 1160–1320 cal CE (95% probability). The burials were earth-cut graves, with bodies placed on their right side following Islamic practice, though there was some variation in orientation (ranging SW–W) [24]. The spatial grouping is interpreted as indicative of family burial areas. Of the 20 dated individuals, 16 were submitted for aDNA analysis.

Agrigento San Leone (SL) (Christian cemetery) (Fig 1. N° 19)

San Leone is located on the left bank of the Akragas (now Fiume San Leone), near its outlet into the Mediterranean Sea. The district is that of the port or emporion of Agrigento and has yielded many maritime-related archaeological finds. In 2014, five amphora burials (enchytrismos) were discovered beneath a thick layer of sand during construction at a house in Viale Viareggio and dated to the

sixth–seventh century CE. The five enchytrismos burials were found in an excavation area about 7.4 by 2.5 meters. The skeletons were contained in amphorae that had been cut, reassembled, bonded with plaster, and laid on a bed of clay and pebbles. Three were oriented with the head to the north and two to the north-west. This burial group is one of three found in the ancient port area. Some graves were located near 7th-century buildings, and others were associated with stone-lined tombs. In some cases, the proximity of buildings of the seventh century was noted and, in one case, amphora burials were associated with other tombs with stone walls and lids. In the archives there are records of a seventh-century amphora reused for burial placed on the crest of a wall bonded with clay and pebbles, situated on an earlier structure, perhaps a storeroom and probably dating to the 4th century [25]. Radiocarbon dating places the human remains between the mid-3rd and mid-6th centuries CE, and three were tested for ancient DNA preservation, unfortunately no aDNA could be used for more sophisticated analyses.

Method details

Radiocarbon dating

This component of the project was led by Derek Hamilton.

AMS laboratory protocol

Sampled human remains were submitted to the SUERC Radiocarbon Laboratory for analysis using accelerator mass spectrometry (AMS) and followed the same methods as presented in Monnereau et al. [26]. Briefly, the remains were pretreated and measured according to the methods outlined by Dunbar et al. [27], and demineralised in 100 mL of 1M HCl at room temperature. Remains were then rinsed to near-neutral pH, immersed in 100 mL of ultrapure MillQ® water, and solubilised fully at ~80ºC before being filtered and freeze-dried. The collagen was graphitised, pressed into aluminium target holders and measured using either the SUERC 5MV tandem or 250kV single-stage AMS. The results are reported

here as conventional radiocarbon ages [28]. All calibrated and modelled date ranges are rounded outward to five years, with modelled dates displayed as italics to differentiate them from the calibrated dates.

Reservoirs, age offsets, and chronological modelling

Radiocarbon measurements can be affected by reservoir effects or the incorporation of 'old' carbon, such as from marine sources or aged materials (i.e., ‘old’ wood effect). When stable isotope data suggest such offsets, correction methods can be applied to the radiocarbon ages or incorporated into Bayesian calibration models. Dietary reservoir effects and Bayesian chronological methods followed the same approach as described in detail within Monnereau et al. [26] for the Segesta cemeteries. Briefly, stable isotope measurements ($\delta^{13}C$) were used to assess the potential for marine reservoir effects. Percent marine protein intake was estimated using linear interpolation between $\delta^{13}C$ end-members for terrestrial herbivores (−20.3‰) and marine fish (−12.8‰), adjusted by +1‰ to account for trophic level enrichment. This yielded dietary end-members of −19.3‰ (fully terrestrial) and −11.8‰ (fully marine). These estimates, assigned an uncertainty of ±10%, were used in OxCal's Mix_Curves function to apply individualised calibration curves combining terrestrial (IntCal20) and marine (Marine20) datasets. A ΔR correction of −111 ± 68 years was applied, based on regional values from the Central Mediterranean using data from the Marine Reservoir Database (http://calib.org/marine/).

Bayesian chronological modelling was conducted using OxCal v4.4, with each burial context treated as an independent bounded phase. In cases where multiple radiocarbon measurements may relate to a single individual, the Combine function was used after applying appropriate marine corrections. Genetic relatedness data were considered but not incorporated into priors, as exploratory models suggested minimal impact on overall chronology.

Ancient DNA analysis

Laboratory methodology

This component of the project was led by Aurore Monnereau, Eleanor Joan Green, Ian Noble, Richard Hagen, Camilla F. Speller and Nathan Wales.

Ancient DNA analysis was conducted at BioArCh, University of York, following protocols the same detailed protocols reported in Monnereau et al. [26]. Briefly, bone surfaces were cleaned by removing an external layer, treated with a 6% sodium hypochlorite solution, rinsed, and UV-irradiated to minimize surface contamination. Approximately 100 mg of bone powder was extracted using a modified silica-spin column method [29] with a double digestion step to maximize endogenous DNA yield [30]. DNA libraries were prepared using a double-stranded Illumina protocol [31] with a unique internal 8 bp barcode ligated to the 5' end of each DNA template as described by Fortes and Paijmans [32]. Libraries were quantified by qPCR, amplified, cleaned with magnetic beads, and assessed for quality. The libraries were grouped into equimolar concentrations for sequencing in single-end mode with a read length of 80 bp on an Illumina HiSeq 4000 platform at the GeoGenetics Sequencing Core.

Some ancient medieval Sicilian individuals with low endogenous content were enriched for mitochondrial DNA using a pre-designed myBaits Expert Mito hybridisation capture kit (Daicel Arbor Biosciences, Ann Arbor, Michigan, USA) according to the manufacturer's protocol (myBaits Manual v4) (S1 Table). Enriched libraries were sequenced on a fraction of the Illumina HiSeq4000 lane at Novogene Corporation (Sacramento, California).

Bioinformatics

This component of the project was led by Aurore Monnereau, Marcela Sadoval-Velasco (African dataset) and Nathan Wales.

Genome mapping. Genome mapping and bioinformatic processing followed the workflow detailed in Monnereau et al. (2024). Illumina adapters and 5' internal barcodes were removed from single-end reads using Cutadapt v3.4 [33], while paired-end reads were merged with PEAR v0.9.11 [34] (minimum overlap 6, p-value 0.01). Trimmed reads were processed through the Paleomix pipeline [35] with default settings, and mapDamage rescaling. Reads were aligned to the hs37d5 reference genome (with mitochondrial DNA replaced by the revised Cambridge reference sequence [36]) using BWA aln [37] (v0.7.17) with disabled seed, minimum mapping quality 30, minimum read length 30, and filtering of unmapped reads. Duplicates were marked with preseq [38]. BAM files were realigned with GATK Indel realigner, and mapDamage 2.0 was used to rescale base quality scores at likely deaminated sites; these rescaled BAM files were used for downstream analyses.

Ancient DNA authentication. Different approaches were used to confirm the authenticity of the data. Firstly, the sequences from blank and library controls were assessed, followed by an investigation into deamination patterns via mapDamage 2.0. The deamination pattern was detected via mapDamage 2.0 [39]. Schmutzi [40] was used to assess the contamination within mtDNA data and the method implemented in ANGSD 0.930 [41] by looking at the contamination through mismatches on the X chromosome in human remains determined to be male (S1 Table). One individual (VGBN6) showed high levels of contamination and was therefore not analysed further in this study.

Uniparental haplogroups. To ascertain the mtDNA haplotypes, the rescaled mtDNA BAM files were processed using the ANGSD 0.930 software [41]. For filtering the bam files, mapping and base quality thresholds above 30 were used, then a FASTA file was created by taking the most common nucleotide for

each position of the mitochondrial reference genome (program option "-doFasta 2"). Thus, the resulting FASTQ file was analysed using a server installation of the Haplogrep V2.2 software [42] with Phylotree build 17 [43]. For further analysis, only those whose haplogroup quality score from Haplogrep was superior to 70% and with an mtDNA contamination level of less than 5% were retained.

For the determination of the Y chromosome haplogroups, the deep shotgun sequencing rescaled BAM files were processed using the default parameters of Yleaf [44] (SI Table S7). The derived terminal SNPs (haplogroup markers) were also checked on the Y haplogroup tree of ISOGG version 2019-2020 (https://isogg.org/tree/).

Genetic sex and kinship analysis. Genetic sex was estimated following the method of Skoglund et al. [45] (S1 Table). Kinship analysis on low-coverage whole-genome data was performed using READ software [46]. To reduce the impact of ancient DNA damage, which disproportionately affects transitions, a panel of 2,217,472 transversion loci was selected from the 1000 Genomes Project [47]. This panel was generated with VCFtools v0.1.15 [48], applying a minor allele frequency cutoff of 0.05 and allowing up to 5% missing data, ensuring a reliable dataset for ancient DNA analyses [49].

Nuclear DNA analysis. The bam files from the article, ancient medieval individuals from Segesta [26] and ancient Sicani individuals [50] were merged to the published datasets compiled by the Allen Ancient DNA Resource database (V50) [51,52]. A pseudo-haploid approach was taken by randomly sampling a read at SNP loci with pileupcaller from the sequenceTools package (source: https://github.com/stschiff/sequenceTools). The convertf program from the EIGENSOFT package [53,54], was used to remove transition sites that may represent post-mortem damage, ensuring the integrity of the dataset. From the Allen Ancient DNA Resource (AADR) database [51,52,55–65], 141 modern populations, 2055 individuals (S2 Table), were incorporated into the analyses. In addition to the AADR database, sub-Saharan African ancestry was investigated using a Sandoval-Velasco et al.'s [66] curated

database of 3,098 individuals from 90 recent populations from sub-Saharan Africa (S3 Table). At least 10,000 transversions SNPs were necessary for an individual to be included in downstream analyses (S1 Table) no evidence of contamination, and no first-degree kinship with other ancient individuals.

Principal Component Analysis. EIGENSOFT [53,54] (with settings: lsqproject: Yes, shrinkmode: Yes, and numoutlieritier:0) was used to access the genetic affinities of medieval Sicilian genomes to modern populations by using smartPCA version 7.2.1. R version 4.0.3 [67] with the package "ggplot2"[68] was used to create PCAs.

Admixture. ADMIXTURE [69] was used to estimate ancestry components in the medieval Sicilian individuals, employing the same reference populations as in the PCA. The dataset was pruned for linkage disequilibrium, and the cross-validation option (-cv) determined the optimal number of ancestral components (K), tested from 1 to 14. Three iterations ensured consistency in the selected cross-validation error. Results were visualized as barplots using R v4.0.3 [67].

Outgroup-F_3-statistic. To formally assess genetic relationships between our medieval Sicilian ancient individuals, test the robustness of non-homogeneous groups, and explore links with burial rites or periods, we conducted outgroup-F_3-statistic using ADMIXTOOLS software [64] by using the option "qp3pop" and "inbreed:YES". The test was performed as F_3(X, Y; Ju|'hoan_North), where X and Y are ancient medieval Sicilian individuals from this study with at least 30,000 transversion SNPs covered on the set 2 panel (1240k) as in [70]. Results were visualized as a heatmap with R v3.6.3 [67] using the package "gplots" v3.1.1 [71].

Expanded Analysis

This component of the project was led by Aurore Monnereau and Nathan Wales.

Relatedness within archaeological sites

Overall, relatively few biologically related individuals were identified within the assemblage as a whole. The lack of relatedness in part stems from the project design, wherein large-scale patterns in genetic diversity were investigated using limited numbers of individuals from single sites, but the lack of detected relatedness may also reflect limitations in DNA preservation in some key sites. For example, at the site of Castello San Pietro (CSP), one of the earliest Islamic cemeteries in Palermo, only three individuals out of thirteen excavated were analysed. None of the three individuals shared the same mitochondrial nor Y-chromosome haplogroups, making it difficult to confirm or refute the hypothesis of a family clan with a eunuch suggested by Di Salvo (2004). Nevertheless, three sites Agrigento (QER), Sant'Agata La Vetere (SAV) and La Villa del Tellaro (TL) individuals with biological relatedness.

Agrigento (QER)

At Agrigento, a Byzantine archaeological site, burials were found within houses. Shared maternal lineages were found within houses suggesting possible nuclear family burials. Indeed, a possible maternal lineage link was found in Tomb 1 Insula I/House I, where two individuals, QERBN30 and QERBN31, share the same maternal haplogroup K1c1. Likewise, QERBN19 and QERBN20 inside Tomb 3 Insula/House II share the same mtDNA mitochondrial haplogroup, T2b7a3. Nuclear DNA preservation was not sufficient to clarify the degree of kinship between pairs of individuals QERBN30 and QERBN31, as well as between QERBN19 and QERBN20. Indeed, for each case, one individual has a coverage lower than 0.1× which is the lowest genome coverage the software READ can analyse (Ralf *et al.* 2018). Thus, only QERBN20 and QERBN31 underwent further analysis.

SampleID	Haplogroup	Rank	Quality	Not found	Found polymorphisms	Remaining

				polymorphisms		polymorphisms
QERBN30	K1c1	1	0.965	498d	73G 146C 152C 263G 750G 1189C 1438G 1811G 2706G 3480G 4769G 7028T 8860G 9055A 9093G 9698C 10398G 10550G 11299C 11377A 11467G 11719A 12308G 12372A 14167T 14766T 14798C 15326G 16224C 16311C	**494A** 3107T 14750G 16519C
QERBN31	K1c1	1	0.9659	498d	73G 146C 152C 263G 750G 1189C 1438G 1811G 2706G 3480G 4769G 7028T 8860G 9055A 9093G 9698C 10398G 10550G 11299C 11377A 11467G 11719A 12308G 12372A 14167T 14766T 14798C 15326G 16224C 16311C	3107C 14750G 16519C

Table A: Haplogrep results for QER_BN30 and QER_BN31. Not found Polys = the polymorphism expected but not found in the samples; Found Polys = polymorphisms used and expected for determining a haplogroup; Remaining Polys = Polymorphisms found but not used to determine a haplogroup as they could be hotspot (e.g., 16519C) or private mutation. In bold are written variants which are found only in one individual.

SampleID	Haplogroup	Rank	Quality	Not found polymorphisms	Found polymorphisms	Remaining polymorphisms
QERBN19	T2b7a3	1	0.9848		73G 263G 709A 750G 930A 1438G 1888A 2706G 4216C 4769G 4917G 5147A 7028T 8697A 8860G 9180G 9966A 10463C 11251G 11440A 11719A 11812G 13368A 14233G 14766T 14905A 15326G 15452A 15607G 15928A 16126C **16292T 16294T 16296T** 16304C	146C 310C 3107C 16519C
QERBN20	T2b7a3	1	0.9504	16292T 16294T 16296T	73G 263G 709A 750G 930A 1438G 1888A 2706G 4216C 4769G 4917G 5147A 7028T 8697A 8860G 9180G 9966A 10463C 11251G 11440A 11719A 11812G 13368A 14233G 14766T 14905A 15326G 15452A 15607G 15928A 16126C 16304C	146C 310C 3107C 16519C

Table B: Haplogrep results for QER_BN19 and QER_BN20. Not found Polys = the polymorphism expected but not found in the samples; Found Polys = polymorphisms used and expected for determining a haplogroup; Remaining Polys = Polymorphisms found but not used to determine a haplogroup as they

could be hotspot (e.g., 16519C) or private mutation. In bold are written variants which are found only in one individual.

Among the Insula/House III, Tomb 3, QERBN28, QERBN6 and QERBN5 share the same mitochondrial haplogroup H3 suggesting a maternal relationship consistent with a hypothesis of a nuclear family by the archaeologists. Specifically, QERBN28 and QERBN6 were identified by READ software as being potentially the same individual or monozygotic twins (Z-upper: 34.284; Z-lower: NA). Therefore, the sequencing data of QERBN28 and QERBN6 were merged and labelled as QERBN6_28. QER_BN5 was not identified by READ software to share a first-degree relationship with those two individuals. As QERBN5 is the earliest individual dating between 715-985 cal CE, whereas QERBN28 and QERBN6 are more recent (775-1000 cal CE and 775-995 cal CE, respectively), they were analysed individually for the nuclear analyses as unrelated individuals: QERBN5 and QERBN6_28. Although it is acknowledged that QERBN5 could be related through a more distant relationship via the maternal side.

SampleID	Haplogroup	Rank	Quality	Not found polymorphisms	Found polymorphisms	Remaining polymorphisms
QERBN6	H3	1	0.9185		263G 750G 1438G 4769G 6776C 8860G 15326G	310C 499A 3107C 3570T **13881T** 16519C
QERBN6	H3	1	0.9185		263G 750G 1438G 4769G 6776C 8860G 15326G	310C 499A 3107C 3570T 16519C
QERBN28	H3	1	0.9185		263G 750G 1438G 4769G 6776C 8860G 15326G	310C 499A 3107C 3570T

						16519C

Table C: Haplogrep results for QER_BN5, QER_BN6 and QER_BN28. Not found Polys = the polymorphism expected but not found in the samples; Found Polys = polymorphisms used and expected for determining a haplogroup; Remaining Polys = Polymorphisms found but not used to determine a haplogroup as they could be hotspot (e.g., 16519C) or private mutation. In bold are written variants which are found only in one individual.

Sant'Agata La Vetere (SAV)

The archaeological site of Sant'Agata La Vetere, on the eastern part of the island, displayed two maternally related individuals, SAVBN2 and SAVBN10. These two individuals share the same mitochondrial haplogroup (W) and are the first-degree kinship related according to READ (Z-upper: 14.029; Z-lower: -19.528). Only SAVBN2 was analysed further in this paper due to the highest number of SNPs present for further analysis.

SampleID	Haplogroup	Rank	Quality	Not found polymorphisms	Found polymorphisms	Remaining polymorphisms
SAVBN2	W	1	0.9307	195C 207A	73G **189G** 204C 263G 709A 750G 1243C 1438G 2706G 3505G 4769G 5046A 5460A 7028T 8251A 8860G 8994A 11674T 11719A 11947G 12414C 12705T 14766T 15326G 15884C 16223T	143A 153G 310C 3107C 13327G 16519C

					16292T	
SAVBN10	W	1	0.9209	189G 195C 207A	73G 204C 263G 709A 750G 1243C 1438G 2706G 3505G 4769G 5046A 5460A 7028T 8251A 8860G 8994A 11674T 11719A 11947G 12414C 12705T 14766T 15326G 15884C 16223T 16292T	143A 153G 310C 3107C 13327G 16519C

Table D: Haplogrep results for SAV_BN2 and SAV_BN10. Not found Polys = the polymorphism expected but not found in the samples; Found Polys = polymorphisms used and expected for determining a haplogroup; Remaining Polys = Polymorphisms found but not used to determine a haplogroup as they could be hotspot (e.g., 16519C) or private mutation. In bold are written variants which are found only in one individual.

La Villa del Tellaro (TL)

La Villa del Tellaro, on the eastern part of the island, only two individuals showed a potential genetic link through the maternal line. TLBN4 and TLBN12 were from the same burial group in different tombs and share the same mtDNA haplogroup J2a2b3. Unfortunately, their nuclear depth of coverage was too low to perform a nuclear kinship analysis with READ software. Due to their low nuclear coverage, neither sample was analysed further.

SampleID	Haplogroup	Rank	Quality	Not found polymorphisms	Found polymorphisms	Remaining polymorphisms
TLBN4	J2a2b3	1	0.9451	4688C 8078A	73G 150T 152C! 195C 263G	3107C 7498A

				16241G	295T 489C 750G 1438G 2706G 4216C 4769G 4802C 6671C 7028T 7476T 8860G 10398G 10499G 11002G 11251G 11377A 11416T 11719A 12570G 12612G 13708A 14766T 15257A 15326G 15452A 15672C 15679G 16069T 16126C	**16559G**
TLBN12	J2a2b3	1	0.9451	4688C 8078A 16241G	73G 150T 152C! 195C 263G 295T 489C 750G 1438G 2706G 4216C 4769G 4802C 6671C 7028T 7476T 8860G 10398G 10499G 11002G 11251G 11377A 11416T 11719A 12570G 12612G 13708A 14766T 15257A 15326G 15452A 15672C 15679G 16069T 16126C	3107C 7498A

Table E: Haplogrep results for TL_BN4 and TL_BN12. Not found Polys = the polymorphism expected but not found in the samples; Found Polys = polymorphisms used and expected for determining a haplogroup; Remaining Polys = Polymorphisms found but not used to determine a haplogroup as they could be hotspot (e.g., 16519C) or private mutation. In bold are written variants which are found only in one individual.

References

1. Lesnes E, Younker RW. San Miceli: Un insediamento rurale paleocristiano nella Sicilia occidentale. Rome: L'Erma di Bretschneider; 2023.

2. Spatafora F. Scavi a Monte Maranfusa (Roccamena, Palermo). Kokalos. 1989;XXXIV-XXXV: 711–718.

3. Spatafora F. Monte Maranfusa/Caltrasi. Guide Breve. Palermo; 2015.

4. Spatafora F. Calatrasi. L'età medievale a Monte Maranfusa. In: Di Stefano CA, Cadei A, editors. Federico e la Sicilia dalla terra alla corona. Palermo: Ediprint; 1995. pp. 162–168.

5. Di Salvo R. I Musulmani della Sicilia occidentale : aspetti antropologici e paleopatologici. Mélanges de l'école française de Rome. 2004;116-1: 389–408. Available: https://www.persee.fr/doc/mefr_1123-9883_2004_num_116_1_8860

6. Isler HP. Monte Iato. In: Di Stefano CA, Cadei A, editors. Federico e la Sicilia dalla terra alla corona. Palermo: Ediprint; 1995. pp. 121–150.

7. Di Salvo R. Gli esemplari di Monte Iato: antropologia e paleopathologia. In: Di Stefano CA, Cadei A, editors. Federico e la Sicilia dalla terra alla corona. Palermo: Ediprint; 1995. pp. 151–162.

8. Maurici F, Alfano A, Muratore S, Salamone F, Scuderi A. Il «Castellazzo» di Monte Iato in Sicilia occidentale (prov. di Palermo). Terza e quarta campagna di scavo. Ricognizioni nel territorio. Fasti On Line Documents & Research. 2014; 317. Available: https://www.fastionline.org/folder/FOLDER-it-2014-317

9. Greco C, Mammina G, Di Salvo R. Necropoli tardoromana in contrada S. Agata (Piana degli Albanesi). In: Di Stefano CA, editor. Di terra in terra : nuove scoperte archeologiche nella provincia di Palermo. Palermo: Museo archeologico regionale; 1991. pp. 161–184.

10. Greco C. Un sito tardoromano sull via Agrigentum-Panormus Scavi nella necropoli in Contrada S. Agata. Kokalos. 1996;XXXIX: 1143– 1163.

11. Bagnera A. From a small town to a capital: The urban evolution of Islamic Palermo (9th–mid-11th century). In: Nef A, editor. A Companion to Medieval Palermo. Leiden, The Netherlands: Brill; 2013. pp. 61–88. doi:10.1163/9789004252530_005

12. Spatafora F, Canzonieri E. Al-Khāliṣa: alcune considerazioni alla luce delle nuove scoperte archeologiche nel Quartiere della Kalsa. In: Nef A, Ardizzone F, editors. Les dynamiques de l'islamisation en méditerranée centrale et en Sicile: nouvelles propositions et découvertes récentes. Rome: Edipuglia; 2014. pp. 233–246.

13. Arcifa L, Bagnera A. Islamizzazione e cultura materiale a Palermo: una riconsiderazione dei contesti ceramici di Castello-San Pietro. In: Nef A, Ardizzone F, editors. Les dynamiques de l'islamisation en méditerranée centrale et en Sicile: nouvelles propositions et découvertes récentes. Rome: Edipuglia; 2014. pp. 165–190.

14. Spatafora F. Nuovi dati preliminari sulla topografia di Palermo in età medievale. Mélanges de l'école française de Rome. 2004;116: 47–78.

15. Battaglia G, La Mantia M, Micciché R, Riolo L. A Norman Age necropoli with a mixed ritual in Palermo. Poster for European Association of Archaeologists conference; 2018.

16. Vassallo S, Nero CA, Battaglia G, Calascibetta G, Chiovaro M, Cucco RM, et al. Attività 2015 della sezione per i beni archeologici della Soprintendenza di Palermo. Notizario Archeologico della Soprintendenza di Palermo. 2016; 9. Available: https://www2.regione.sicilia.it/beniculturali/dirbenicult/notiziarioarcheologicopalermo/09_Attivita_UO5_5.pdf

17. Carver M, Molinari A, Aniceti V, Capelli C, Colangeli F, Drieu L, et al. SICILY IN TRANSITION: New research on early medieval Sicily, 2017-2018. Fasti On Line Documents & Research. 2019; 437. Available: https://www.fastionline.org/folder/FOLDER-it-2019-437

18. Parello MC, Rizzo MS. Agrigentum ed il suo territorio in età tardo antica. In: Álvarez Martínez JM, Nogales Basarrate T, Rodà de Llanza I, editors. ACTAS XVIII Congreso Internacionali Arqueologia Clasica, Centro y periferia en el mundo clasico. Mérida: Museo Nacional de Arte Romano; 2014. pp. 1823–1826.

19. Di Giuseppe Z. Le tombe del Quartiere Ellenistico Romano, campagna di scavo 2013. In: Parello MC, Rizzo MS, editors. Agrigento Romana, Scavi e Ricerche nel Quartiere Ellenistico Romano, Campagna 2013. Palermo: Regione Siciliana, Assessorato dei beni culturali e dell'identità siciliana; 2015. pp. 89–110.

20. Falzone G. L'Ipogeo "P" e la Longue Durée di un edificio funerario tardoantico. In: Parello MC, Rizzo MS, editors. Paesaggi Urbani Tardoantichi Casa a Confronto. Bari: Edipuglia; 2016. pp. 175–184.

21. Fanelli R. Il Quartiere Ellenistico-Romano tra tardoantico e altomedioevo: le indagini archeoantropologiche del 2014. In: Parello MC, Rizzo MS, editors. Paesaggi urbani tardoantichi Casi a confront. Bari: Edipuglia; 2016. pp. 353–358.

22. Nicoletti R, Carver M. Enna. In: Carver M, editor. Remembering the Dead in Medieval Sicily. Florence: All'Insegna del giglio;

23. Patané A, Tanasi D, Cali D. Indagini archeologiche a Sant'Agata la Vetere e Sant'Agata al Carcere. In: Branciforti MG, La Rosa V, editors. Tra Lava e mare: Contributi all'archaiologhia di Catania. Catania: Le Nove Muse Editrice; 2010. pp. 337–354.

24. Garipoli S. Nuovi dati sui cimiteri di rito islamico in Sicilia: il gruppo umano del cimitero di Contrada Caddeddi (Noto). Cronache di Archeologia. 2018;37: 435–448.

25. Caminneci V. Enchytrismos. Seppellire in vaso nell'antica Agrigento. In: Caminneci V, editor. Parce sepulto: il rito e la morte tra passato e presente. Palermo: Regione Siciliana; 2012. pp. 111–134.

26. Monnereau A, Ughi A, Orecchioni P, Hagan R, Talbot HM, Nikita E, et al. Multi-proxy bioarchaeological analysis of skeletal remains shows genetic discontinuity in a Medieval Sicilian community. R Soc Open Sci. 2024;11: 240436. doi:10.1098/rsos.240436

27. Dunbar E, Cook GT, Naysmith P, Tripney BG, Xu S. AMS ^{14}C Dating at the Scottish Universities Environmental Research Centre (SUERC) Radiocarbon Dating Laboratory. Radiocarbon. 2016;58: 9–23. doi:10.1017/RDC.2015.2

28. Stuiver M, Polach HA. Discussion Reporting of ^{14}C Data. Radiocarbon. 1977;19: 355–363. doi:10.1017/S0033822200003672

29. Yang DY, Eng B, Waye JS, Dudar JC, Saunders SR. Technical note: improved DNA extraction from ancient bones using silica-based spin columns. Am J Phys Anthropol. 1998;105: 539–543. doi:10.1002/(SICI)1096-8644(199804)105:4<539::AID-AJPA10>3.0.CO;2-1

30. McGrath K, Rowsell K, St-Pierre CG, Tedder A, Foody G, Roberts C, et al. Identifying Archaeological Bone via Non-Destructive ZooMS and the Materiality of Symbolic Expression: Examples from Iroquoian Bone Points. Sci Rep. 2019;9: 11027. doi:10.1038/s41598-019-47299-x

31. Meyer M, Kircher M. Illumina sequencing library preparation for highly multiplexed target capture and sequencing. Cold Spring Harb Protoc. 2010;2010: db.prot5448. doi:10.1101/pdb.prot5448

32. Fortes GG, Paijmans JLA. Analysis of Whole Mitogenomes from Ancient Samples. Methods Mol Biol. 2015;1347: 179–195. doi:10.1007/978-1-4939-2990-0_13

33. Martin M. Cutadapt removes adapter sequences from high-throughput sequencing reads. EMBnet.journal.

2011;17: 10–12. doi:10.14806/ej.17.1.200

34. Zhang J, Kobert K, Flouri T, Stamatakis A. PEAR: a fast and accurate Illumina Paired-End reAd mergeR. Bioinformatics. 2014;30: 614–620. doi:10.1093/bioinformatics/btt593

35. Schubert M, Ermini L, Der Sarkissian C, Jónsson H, Ginolhac A, Schaefer R, et al. Characterization of ancient and modern genomes by SNP detection and phylogenomic and metagenomic analysis using PALEOMIX. Nat Protoc. 2014;9: 1056–1082. doi:10.1038/nprot.2014.063

36. Andrews RM, Kubacka I, Chinnery PF, Lightowlers RN, Turnbull DM, Howell N. Reanalysis and revision of the Cambridge reference sequence for human mitochondrial DNA. Nat Genet. 1999;23: 147. doi:10.1038/13779

37. Li H, Durbin R. Fast and accurate short read alignment with Burrows–Wheeler transform. Bioinformatics. 2009;25: 1754–1760. doi:10.1093/bioinformatics/btp324

38. Daley T, Smith AD. Modeling genome coverage in single-cell sequencing. Bioinformatics. 2014;30: 3159–3165. doi:10.1093/bioinformatics/btu540

39. Jónsson H, Ginolhac A, Schubert M, Johnson PLF, Orlando L. mapDamage2.0: fast approximate Bayesian estimates of ancient DNA damage parameters. Bioinformatics. 2013;29: 1682–1684. doi:10.1093/bioinformatics/btt193

40. Renaud G, Slon V, Duggan AT, Kelso J. Schmutzi: estimation of contamination and endogenous mitochondrial consensus calling for ancient DNA. Genome Biol. 2015;16: 224. doi:10.1186/s13059-015-0776-0

41. Korneliussen TS, Albrechtsen A, Nielsen R. ANGSD: Analysis of Next Generation Sequencing Data. BMC Bioinformatics. 2014;15: 356. doi:10.1186/s12859-014-0356-4

42. Weissensteiner H, Pacher D, Kloss-Brandstätter A, Forer L, Specht G, Bandelt H-J, et al. HaploGrep 2: mitochondrial haplogroup classification in the era of high-throughput sequencing. Nucleic Acids Res. 2016;44: W58–63. doi:10.1093/nar/gkw233

43. van Oven M. PhyloTree Build 17: Growing the human mitochondrial DNA tree. Forensic Science International: Genetics Supplement Series. 2015;5: e392–e394. doi:10.1016/j.fsigss.2015.09.155

44. Ralf A, González DM, Zhong K, Kayser M. Yleaf: Software for Human Y-Chromosomal Haplogroup Inference from Next-Generation Sequencing Data. Molecular Biology and Evolution. 2018. pp. 1820–1820. doi:10.1093/molbev/msy080

45. Skoglund P, Storå J, Götherström A, Jakobsson M. Accurate sex identification of ancient human remains using DNA shotgun sequencing. J Archaeol Sci. 2013;40: 4477–4482. doi:10.1016/j.jas.2013.07.004

46. Monroy Kuhn JM, Jakobsson M, Günther T. Estimating genetic kin relationships in prehistoric populations. PLoS One. 2018;13: e0195491. doi:10.1371/journal.pone.0195491

47. 1000 Genomes Project Consortium, Auton A, Brooks LD, Durbin RM, Garrison EP, Kang HM, et al. A global reference for human genetic variation. Nature. 2015;526: 68–74. doi:10.1038/nature15393

48. Danecek P, Auton A, Abecasis G, Albers CA, Banks E, DePristo MA, et al. The variant call format and VCFtools. Bioinformatics. 2011;27: 2156–2158. doi:10.1093/bioinformatics/btr330

49. Nakatsuka N, Harney É, Mallick S, Mah M, Patterson N, Reich D. ContamLD: estimation of ancient nuclear DNA contamination using breakdown of linkage disequilibrium. Genome Biol. 2020;21: 199. doi:10.1186/s13059-020-02111-2

50. Reitsema LJ, Mittnik A, Kyle B, Catalano G, Fabbri PF, Kazmi ACS, et al. The diverse genetic origins of a Classical period Greek army. Proc Natl Acad Sci U S A. 2022;119: e2205272119.

doi:10.1073/pnas.2205272119

51. Mallick S, Micco A, Mah M, Ringbauer H, Lazaridis I, Olalde I, et al. The Allen Ancient DNA Resource (AADR) a curated compendium of ancient human genomes. Sci Data. 2024;11: 182. doi:10.1038/s41597-024-03031-7

52. Mallick S, Reich D. The Allen Ancient DNA Resource (AADR): A curated compendium of ancient human genomes. Harvard Dataverse; 2023. doi:10.7910/DVN/FFIDCW

53. Patterson N, Price AL, Reich D. Population structure and eigenanalysis. PLoS Genet. 2006;2: e190. doi:10.1371/journal.pgen.0020190

54. Price AL, Patterson NJ, Plenge RM, Weinblatt ME, Shadick NA, Reich D. Principal components analysis corrects for stratification in genome-wide association studies. Nat Genet. 2006;38: 904–909. doi:10.1038/ng1847

55. Jeong C, Balanovsky O, Lukianova E, Kahbatkyzy N, Flegontov P, Zaporozhchenko V, et al. The genetic history of admixture across inner Eurasia. Nat Ecol Evol. 2019;3: 966–976. doi:10.1038/s41559-019-0878-2

56. Skoglund P, Thompson JC, Prendergast ME, Mittnik A, Sirak K, Hajdinjak M, et al. Reconstructing Prehistoric African Population Structure. Cell. 2017;171: 59–71.e21. doi:10.1016/j.cell.2017.08.049

57. Vyas DN, Al-Meeri A, Mulligan CJ. Testing support for the northern and southern dispersal routes out of Africa: an analysis of Levantine and southern Arabian populations. Am J Phys Anthropol. 2017;164: 736–749. doi:10.1002/ajpa.23312

58. Lazaridis I, Nadel D, Rollefson G, Merrett DC, Rohland N, Mallick S, et al. Genomic insights into the origin of farming in the ancient Near East. Nature. 2016;536: 419–424. doi:10.1038/nature19310

59. Mallick S, Li H, Lipson M, Mathieson I, Gymrek M, Racimo F, et al. The Simons Genome Diversity Project: 300 genomes from 142 diverse populations. Nature. 2016;538: 201–206. doi:10.1038/nature18964

60. Mondal M, Casals F, Xu T, Dall'Olio GM, Pybus M, Netea MG, et al. Genomic analysis of Andamanese provides insights into ancient human migration into Asia and adaptation. Nat Genet. 2016;48: 1066–1070. doi:10.1038/ng.3621

61. Skoglund P, Posth C, Sirak K, Spriggs M, Valentin F, Bedford S, et al. Genomic insights into the peopling of the Southwest Pacific. Nature. 2016;538: 510–513. doi:10.1038/nature19844

62. Lazaridis I, Patterson N, Mittnik A, Renaud G, Mallick S, Kirsanow K, et al. Ancient human genomes suggest three ancestral populations for present-day Europeans. Nature. 2014;513: 409–413. doi:10.1038/nature13673

63. Meyer M, Kircher M, Gansauge M-T, Li H, Racimo F, Mallick S, et al. A high-coverage genome sequence from an archaic Denisovan individual. Science. 2012;338: 222–226. doi:10.1126/science.1224344

64. Patterson N, Moorjani P, Luo Y, Mallick S, Rohland N, Zhan Y, et al. Ancient admixture in human history. Genetics. 2012;192: 1065–1093. doi:10.1534/genetics.112.145037

65. Pickrell JK, Patterson N, Barbieri C, Berthold F, Gerlach L, Güldemann T, et al. The genetic prehistory of southern Africa. Nat Commun. 2012;3: 1143. doi:10.1038/ncomms2140

66. Sandoval-Velasco M, Jagadeesan A, Ramos-Madrigal J, Ávila-Arcos MC, Fortes-Lima CA, Watson J, et al. The ancestry and geographical origins of St Helena's liberated Africans. Am J Hum Genet. 2023;110: 1590–1599. doi:10.1016/j.ajhg.2023.08.001

67. R Core Team. R: A Language and Environment for Statistical Computing. Vienna, Austria: R Foundation for Statistical Computing; 2020. Available: https://www.R-project.org/

68. Wickham H. ggplot2: Elegant Graphics for Data Analysis. Springer-Verlag New York; 2016. Available: https://ggplot2.tidyverse.org

69. Alexander DH, Novembre J, Lange K. Fast model-based estimation of ancestry in unrelated individuals. Genome Res. 2009;19: 1655–1664. doi:10.1101/gr.094052.109

70. Fu Q, Posth C, Hajdinjak M, Petr M, Mallick S, Fernandes D, et al. The genetic history of Ice Age Europe. Nature. 2016;534: 200–205. doi:10.1038/nature17993

71. Warnes GR, Bolker B, Bonebakker L, Gentleman R, Huber W, Liaw A, et al. gplots: Various R Programming Tools for Plotting Data. 2020 [cited 10 Mar 2021]. Available: https://cran.r-project.org/package=gplots
