# Supplementary material for: Genetic histories of individuals from multi-faith medieval Sicily
Source: PLoS One. 2026 Jun 24;21(6):e0350298. doi: 10.1371/journal.pone.0350298 (PMC13293407; doi:10.1371/journal.pone.0350298)
Supplement: S2 File — Information regarding the ethical, cultural and scientific considerations specific to inclusivity in global research. (PDF) [file pone.0350298.s002.pdf]

# Inclusivity in global research

PLOS' policy on inclusivity in global research aims to improve transparency in the reporting of research performed outside of researchers' own country or community and ensures that PLOS publications reporting global research adhere to high standards for research ethics and authorship. Authors of relevant research articles may be asked to complete the questionnaire below, which outlines ethical, cultural, and scientific considerations specific to inclusivity in global research. This questionnaire may be requested when researchers have travelled to a different country to conduct research, if research uses samples collected in another country, research with Indigenous populations or their lands, or if research is on cultural artefacts. Researchers travelling to another country solely to use laboratory equipment will not normally be required to complete the questionnaire. However, the questionnaire can be requested at the journal's discretion for any submission – if you have been requested to complete this questionnaire by the PLOS journal you submitted to, please do so.

Please complete the questionnaire below and include this as a Supporting Information file with your manuscript. Note that if your paper is accepted for publication, this checklist will be published with your article in the supporting information files. Please ensure that you reference the checklist in the main body of your manuscript. We suggest adding a subsection 'Inclusivity in global research' to your Methods section and adding the following sentence: "Additional information regarding the ethical, cultural, and scientific considerations specific to inclusivity in global research is included in the Supporting Information (SX Checklist)"

The questions have been designed to be applicable to a wide range of study types, and there are subsections for both human subjects research and non-human subjects research. If any of the questions are not relevant to your research please mark them as "N/A" as appropriate.

## Ethical considerations, permits and authorship

*This section is applicable to all research types.*

Provide details as to who granted permissions and/or consent for the study to take place in the Methods section of your manuscript. This should include the names of **all** ethics boards, governmental organizations, community leaders or other bodies that provided approval for the study. If individuals provided approval refer to these people by their role or title but do not list their name(s).

| |
|---|
| Reported on page number: 24<br><br>We have updated the beginning of the methods section with these details. Specifically, we have written: "All necessary permits were obtained for the described study, which complied with all relevant regulations. Permission for destructive sampling were granted by the Soprintendenza di Enna, Museo Archeologico Antonino Salinas Palermo, Museo Regionale Paolo Orsi Siracusa, Parco Archeologico della Valle dei Templi |

Agrigento, Soprintendenza di Palermo, Soprintendenza di Trapani and Università di Catania. Further details on the authorisations, permit numbers and repositories are provided in S1 Table."

If there were any deviations from the study protocol after approval was obtained please provide details of these changes in the Methods section of your manuscript.

We did not deviate from the study protocol after the approval.

Did this study involve local collaborators that are residents of the country where the research was conducted or members of the community studied? If you do not have any authors from said communities, please provide an explanation for this below.

Yes, this study involves many local/italian researchers. These stakeholders were consulted throughout the experimental design, experiments and interpretations. While it is not possible to name all of these archaeologists and researchers who provided samples, two authors on this work coordinated the research in Sicily. Both Dr Alessandra Molinari and Dr Paola Orecchioni are Italian citizens and work in the country where the research is focused.

Everyone listed as an author should meet PLOS' criteria for authorship and all individuals who meet these criteria should be included in the author byline, rather than the acknowledgements. For further information please see the journal's Authorship Policy.

## Human subjects research (e.g. health research, medical research, cross-cultural psychology)

Did you obtain written informed consent from a representative of the local community or region before the research took place? How did you establish who speaks for the community? Details of written informed consent obtained from study participants should be reported separately in the Methods section of your manuscript.

This project is focused on archaeological questions and includes individuals from medieval contexts. For that reason, written consent cannot be obtained from people who are unambiguously part of a descendant community. That said, all regulations were followed to ensure proper sampling of archaeological remains and the local communities were aware of the archaeological science project.

How did members of the local community provide input on the aims of the research investigation, its methodology, and its anticipated outcome(s)?

| |
|---|
| Local researchers and archaeologists provided invaluable knowledge about the archaeological sites and interpretations of the excavated graves. The wider SICTRANSIT project also actively engaged with the local community in Sicily through in-person activities and online outreach. |

When engaging with the local community, how did you ensure that the informed consent documents and other materials could be understood by local stakeholders?

| |
|---|
| This project did not include informed consent, but other outreach materials were carefully designed to target a wide audience, including individuals that may not have much exposure to archaeology or scientific methods. As stated below, members of our team visited local schools and other public venues where members of the public could hear directly from archaeologists and ask questions to learn more about the history of Sicily. |

Will the findings of the research be made available in an understandable format to stakeholders in the community where the study was conducted (e.g. via a presentation, summary report, copies of publications, etc.)? Please provide details of how this will be achieved.

| |
|---|
| Indeed, a significant effort has been made to inform the public about the project and our results. This form of outreach is generally expected for archaeological research and the SICTRANSIT project has excelled in informing stakeholders about ongoing results.<br><br>Archaeological results were shared with the local Sicilian communities every year, numerous outreach activities done in person as well as online. For example, the research associated with the site of Castronovo was exhibited to local audiences with talks in the town centre at the start and end of each season. The locals were invited to participate in discussions, school visits and site tours, as well as learning more through a public-facing website that includes both English and Italian content:<br>https://sicily-in-transition.org<br><br>The DNA-focused results of this work will be broadcast to a wide audience through our media relation teams once this article is published. |

**Non-human subjects research using specimens/ animals collected as part of the study, or those housed in archival collections. Examples include archaeology, paleontology, botany and zoology.**

Did the permission you obtained from a local authority to perform the study include an agreement on access to outputs and benefit sharing? This may include procedures to enable fair distribution of the benefits and resources arising from the research performed. Please include any details of Prior Informed Consent and Benefit Sharing Agreements obtained. These may be required by field-specific regulations, for example the Convention on Biological Diversity (CBD) and the associated Nagoya Protocol.

This project used archaeological samples as described above. All the specimens described in this article are from human remains and the necessary permissions were taken, as provided above. In short, all necessary regulations were followed and we obtained permissions from all relevant governing bodies. The outputs of this project, such as publications, will be described to the local community through our outreach efforts and further benefited by the Open Access nature of the journal.

If the material used in your study was imported, please A) provide the year it was imported and B) indicate whether permits were obtained to import/export the materials used, C) provide details of any permits obtained. If this information is not available, please indicate this.

Archaeological human remains were collected in Italy with the necessary permits by our Italian colleagues and shipped to the United Kingdom. York's Department of Archaeology holds necessary permits for the processing of archaeological material and no special permits were required for importing the skeletal remains. In the UK, regulations regarding human remains are restricted to the past 100 years, thus the Sicilian human remains are out of scope for special permits.

The material was shipped from Italy to the UK in 2017-2021.

If you used archival specimens, please state how the material used in your study was acquired by the institute it is held in and provide details of any permits obtained for the original excavations/ sample collection. If this information is not available, please indicate this.

A majority of the human remains were excavated before the SICTRANSIT project was developed. Archaeological bones have been stored in multiple repositories in Sicily, each with their own system of archiving and cataloging remains. These repositories provided sample authorisations for destructive testing,

but we do not have additional details on the original sample collection permits. Nonetheless, each repository is a well known museum or *Soprintendenza* (i.e. official archaeological curator for a region of Italy), and thus they are entities that carefully monitor the origins of their collections and ensure that further archaeological research is performed properly.

How was the potential cultural significance of the materials collected in your study to local communities considered in your research design? Were Indigenous peoples and/or local researchers and institutions involved with archaeological excavations / collection of specimens? If so, please provide a description of their involvement.

The cultural significance of the archaeological sites and the human remains was carefully considered during the research design, and local Italian researchers were involved in the selection of sites and materials. We were particularly cautious about the testing of cemeteries which demonstrate different religious practices, and have gone to great lengths that the phrasing in our article accurately captures this sensitivity. Local people were involved in numerous discussions about the sites and history of Sicily, and we will share the publication of this article on social media and other outlets to ensure these communities understand how scientific analysis of the human remains has improved our understanding of medieval communities in Sicily.

If your manuscript includes photographs of human remains please indicate whether authors obtained permission from descendants or affiliated cultural communities to do so.

The manuscript does not include photographs of human remains.
