# Supplementary material for: Genetic histories of individuals from multi-faith medieval Sicily
Source: PLoS One. 2026 Jun 24;21(6):e0350298. doi: 10.1371/journal.pone.0350298 (PMC13293407; doi:10.1371/journal.pone.0350298)
Supplement: Attachment — Submitted filename: Response to Reviewers.pdf [file pone.0350298.s007.pdf]

Dear Academic editor Marta Maria Ciucani and Reviewers,

On behalf of all of the co-authors, we would like to thank you for the opportunity to revise our manuscript so that it can be considered for publication in *PLOS ONE*. Likewise, we would like to express our gratitude to the two reviewers for their comments and suggestions which have improved the quality and clarity of our work. Below, we provide a detailed, point-by-point response to each comment raised by the reviewers, along with the necessary changes for submitting to the journal. All changes made in the revised manuscript are written here in **green**. We hope the reviewers agree that their recommendations have helped to turn the manuscript into a stronger and more well-rounded piece of work.

Kind regards,
Aurore Monnereau and Nathan Wales

Journal Requirements:

When submitting your revision, we need you to address these additional requirements.

1. Please ensure that your manuscript meets PLOS ONE's style requirements, including those for file naming. The PLOS ONE style templates can be found at

https://journals.plos.org/plosone/s/file?id=wjVg/PLOSOne_formatting_sample_main_body.pdf and

https://journals.plos.org/plosone/s/file?id=ba62/PLOSOne_formatting_sample_title_authors_affiliations.pdf

Response: To the Academic Editor and both Reviewers, we greatly appreciate all of your efforts to help improve our manuscript. As you see here, we have used this document to respond to each comment, with our responses in **GREEN** to distinguish them from the questions and hyperlinks.

Regarding the formatting, we have now updated the manuscript to meet PLOS ONE's style requirements. In particular, the first page has been updated with the specified title font and author affiliation addresses, the heading formats have been updated to your required fonts, the figure captions have been set within the text, and the naming strategy for the supplemental documents has been changed. We have also updated our supporting text document to have the same formatting. If you notice that we have somehow overlooked other requirements, please let us know so that we can rectify the issues.

2. Please include a complete copy of PLOS' questionnaire on inclusivity in global research in your revised manuscript. Our policy for research in this area aims to improve transparency in the reporting of research performed outside of researchers' own country or community. The policy applies to researchers who have travelled to a different country to conduct research, research with Indigenous populations or their lands, and research on cultural artefacts. The

questionnaire can also be requested at the journal's discretion for any other submissions, even if these conditions are not met. Please find more information on the policy and a link to download a blank copy of the questionnaire here: https://journals.plos.org/plosone/s/best-practices-in-research-reporting. Please upload a completed version of your questionnaire as Supporting Information when you resubmit your manuscript.

Response: We have completed this questionnaire and are attaching it with the submission.

3. In your manuscript, please provide additional information regarding the specimens used in your study. Ensure that you have reported human remain specimen numbers and complete repository information, including museum name and geographic location.

If permits were required, please ensure that you have provided details for all permits that were obtained, including the full name of the issuing authority, and add the following statement:

> 'All necessary permits were obtained for the described study, which complied with all relevant regulations.'

If no permits were required, please include the following statement:

> 'No permits were required for the described study, which complied with all relevant regulations.'

Response: Thank you for providing this guidance. We have moved this statement from our previous "ethics statement" section to the beginning of the methods. The requested details have been added to Table S1: geographic location (using latitude/longitude coordinates), museum/repository name, and the entity that provided permission for analysing each individual.

Regarding the point about "human remain specimen numbers" we have provided the full information for how these repositories organise their collections. Specifically, the remains are organised by the Tomb number, and where necessary, an additional specification of an individual in a tomb. We have reported this information in Table S1; for example, individual "CDMBN1" is from the Corso dei Mille site with reference information "Tomb C144/Ind6".

For more information on PLOS One's requirements for paleontology and archeology research, see https://journals.plos.org/plosone/s/submission-guidelines#loc-paleontology-and-archaeology-research.

4. We note that the grant information you provided in the 'Funding Information' and 'Financial Disclosure' sections do not match.

When you resubmit, please ensure that you provide the correct grant numbers for the awards you received for your study in the ‘Funding Information’ section.

Response: We have tried to update the financial information, but we have so far not been able to locate a section in the submission called “Funding information”. So it is somewhat unclear to us which pieces do not match. There was a section in the first submission of our manuscript, but this has been removed in the revision to meet your guidelines. Hopefully this is identified and corrected as we complete the online portion of the submission.

5. Thank you for stating the following financial disclosure:

“This work was made possible through funding from the European Research Council (ERC), awarded under the Horizon 2020 research and innovation programme (Grant Agreement No. 693600, ‘The Archaeology Of Regime Change: Sicily In Transition’ (SICTRANSIT)).”

Please state what role the funders took in the study. If the funders had no role, please state: "The funders had no role in study design, data collection and analysis, decision to publish, or preparation of the manuscript."

If this statement is not correct you must amend it as needed.

Please include this amended Role of Funder statement in your cover letter; we will change the online submission form on your behalf.

Response: This amended Role of Funder statement has been provided in the cover letter and repeated here:

> This work was made possible through funding from the European Research Council (ERC), awarded under the Horizon 2020 research and innovation programme (Grant Agreement No. 693600, ‘The Archaeology Of Regime Change: Sicily In Transition’ (SICTRANSIT)). The funders had no role in study design, data collection and analysis, decision to publish, or preparation of the manuscript.

Thank you for helping change it on our behalf.

6. Please note that funding information should not appear in any section or other areas of your manuscript. We will only publish funding information present in the Funding Statement section of the online submission form. Please remove any funding-related text from the manuscript.

Response: This section of the manuscript has been removed.

7. Please note that your Data Availability Statement is currently missing a direct link to access each database. If your manuscript is accepted for publication, you will be asked to provide these details on a very short timeline. We therefore suggest that you provide this information now, though we will not hold up the peer review process if you are unable.

Response: We have now added a direct link for our raw DNA data in the Data Availability Statement.

8. Please provide a complete Data Availability Statement in the submission form, ensuring you include all necessary access information or a reason for why you are unable to make your data freely accessible. If your research concerns only data provided within your submission, please write "All data are in the manuscript and/or supporting information files" as your Data Availability Statement.

Response: We have updated the Data Availability Statement as following:

> The raw DNA sequencing files are available in NCBI Project PRJNA1400023. The dataset is publicly available at https://www.ncbi.nlm.nih.gov/sra/PRJNA1400023. All other data are in the manuscript and supporting files (S1 File, S1 Table, S2 Table, S3 Table).

We note that the NCBI link is not yet active, but we will set the data to release at the same time as the publication of this manuscript.

9. We note that Figures 1, 4, and 5 in your submission contain map images which may be copyrighted. All PLOS content is published under the Creative Commons Attribution License (CC BY 4.0), which means that the manuscript, images, and Supporting Information files will be freely available online, and any third party is permitted to access, download, copy, distribute, and use these materials in any way, even commercially, with proper attribution. For these reasons, we cannot publish previously copyrighted maps or satellite images created using proprietary data, such as Google software (Google Maps, Street View, and Earth). For more information, see our copyright guidelines: http://journals.plos.org/plosone/s/licenses-and-copyright.

We require you to either (1) present written permission from the copyright holder to publish these figures specifically under the CC BY 4.0 license, or (2) remove the figures from your submission:

> 1. You may seek permission from the original copyright holder of Figure(s) [#] to publish the content specifically under the CC BY 4.0 license.

We recommend that you contact the original copyright holder with the Content Permission Form (http://journals.plos.org/plosone/s/file?id=7c09/content-permission-form.pdf) and the following text:

“I request permission for the open-access journal PLOS ONE to publish XXX under the Creative Commons Attribution License (CCAL) CC BY 4.0 (http://creativecommons.org/licenses/by/4.0/). Please be aware that this license allows unrestricted use and distribution, even commercially, by third parties. Please reply and provide explicit written permission to publish XXX under a CC BY license and complete the attached form.”

Please upload the completed Content Permission Form or other proof of granted permissions as an "Other" file with your submission.

In the figure caption of the copyrighted figure, please include the following text: “Reprinted from [ref] under a CC BY license, with permission from [name of publisher], original copyright [original copyright year].”

> 2. If you are unable to obtain permission from the original copyright holder to publish these figures under the CC BY 4.0 license or if the copyright holder’s requirements are incompatible with the CC BY 4.0 license, please either i) remove the figure or ii) supply a replacement figure that complies with the CC BY 4.0 license. Please check copyright information on all replacement figures and update the figure caption with source information. If applicable, please specify in the figure caption text when a figure is similar but not identical to the original image and is therefore for illustrative purposes only.

The following resources for replacing copyrighted map figures may be helpful:

USGS National Map Viewer (public domain): http://viewer.nationalmap.gov/viewer/

The Gateway to Astronaut Photography of Earth (public domain): http://eol.jsc.nasa.gov/sseop/clickmap/

Maps at the CIA (public domain): https://www.cia.gov/library/publications/the-world-factbook/index.html and https://www.cia.gov/library/publications/cia-maps-publications/index.html

NASA Earth Observatory (public domain): http://earthobservatory.nasa.gov/

Landsat: http://landsat.visibleearth.nasa.gov/

USGS EROS (Earth Resources Observatory and Science (EROS) Center) (public domain): http://eros.usgs.gov/#

Natural Earth (public domain): http://www.naturalearthdata.com/

Response: All of the figures which we have included the manuscript were created by us using R software and the Natural Earth public domain data. The captions for Figure 1 (map of Sicily) and Figure 4 (inset map of Africa) have been updated to explain which R libraries were used to create each map. Therefore, there should be no issue related to copyrighted material.

We note that your comment also refers to Figure 5, but this figure does not include a map. Rather, it is a PCA which displays genetic distances between individuals.

10. Please upload a new copy of Figure 5 as the detail is not clear. Please follow the link for more information: https://journals.plos.org/plosone/s/figures

Response: We’ve used your NAAS software to convert our high resolution image to a new TIF file. The website reports that now the “Image complies with specification.” That said, we note that the figure still gets blurry when zoomed in close, so we could provide another format (e.g. PDF, PNG) if your team would rather work with it.

11. Please include captions for your Supporting Information files at the end of your manuscript, and update any in-text citations to match accordingly. Please see our Supporting Information guidelines for more information: http://journals.plos.org/plosone/s/supporting-information.

Response: We have updated it and wrote the captions for the supporting information files at the end of the manuscript.

12. We note that there is identifying data in the Supporting Information files:

- Supplementary_Table_S1_Dataset.xlsx
- Supplementary_Table_S2_Dataset.xlsx
- Supplementary_Table_S3_Dataset.xlsx

Due to the inclusion of these potentially identifying data, we have removed this file from your file inventory. Prior to sharing human research participant data, authors should consult with an ethics committee to ensure data are shared in accordance with participant consent and all applicable local laws.

Data sharing should never compromise participant privacy. It is therefore not appropriate to publicly share personally identifiable data on human research participants. The following are examples of data that should not be shared:

-Name, initials, physical address

-Ages more specific than whole numbers

-Internet protocol (IP) address

-Specific dates (birth dates, death dates, examination dates, etc.)

-Contact information such as phone number or email address

-Location data

-ID numbers that seem specific (long numbers, include initials, titled “Hospital ID”) rather than random (small numbers in numerical order)

Data that are not directly identifying may also be inappropriate to share, as in combination they can become identifying. For example, data collected from a small group of participants, vulnerable populations, or private groups should not be shared if they involve indirect

identifiers (such as sex, ethnicity, location, etc.) that may risk the identification of study participants.

Additional guidance on preparing raw data for publication can be found in our Data Policy (https://journals.plos.org/plosone/s/data-availability#loc-human-research-participant-data-and-other-sensitive-data) and in the following article: http://www.bmj.com/content/340/bmj.c181.long.

Please remove or anonymize all personal information (Ages more specific than whole numbers and Location data), ensure that the data shared are in accordance with participant consent, and re-upload a fully anonymized data set. Please note that spreadsheet columns with personal information must be removed and not hidden as all hidden columns will appear in the published file.

Response: The supporting information files contain no personal information on the human remains. In the S1 Table, we have the ID number for each archaeology individual, but there is no personal identification information for individuals who died many centuries ago and with no known descendent.

The S2 Table is only a list of populations used for comparison with our data, with no information about the individuals themselves. The genomic data for these populations has been published in a range of articles cited within the table.

We have changed the format of the S3 Table so that it is consistent with the S2 Table; only information at the population level can be found. And as with the S2 Table, this genomic data of individuals from Africa has been published in a journal that works carefully to ensure individuals are anonymised. For your reference, this reference dataset is from this source:

Sandoval-Velasco M, Jagadeesan A, Ramos-Madrigal J, Ávila-Arcos MC, Fortes-Lima CA, Watson J, et al. The ancestry and geographical origins of St Helena’s liberated Africans. *Am J Hum Genet*. 2023;110: 1590–1599. doi:10.1016/j.ajhg.2023.08.001

13. If the reviewer comments include a recommendation to cite specific previously published works, please review and evaluate these publications to determine whether they are relevant and should be cited. There is no requirement to cite these works unless the editor has indicated otherwise.

Response: The reviewers did not ask us to include a specific source to cite.

14. Please review your reference list to ensure that it is complete and correct. If you have cited papers that have been retracted, please include the rationale for doing so in the manuscript text, or remove these references and replace them with relevant current references. Any changes to the reference list should be mentioned in the rebuttal letter that accompanies your revised manuscript. If you need to cite a retracted article, indicate the

article's retracted status in the References list and also include a citation and full reference for the retraction notice.

Review Comments to the Author

Please use the space provided to explain your answers to the questions above. You may also include additional comments for the author, including concerns about dual publication, research ethics, or publication ethics. (Please upload your review as an attachment if it exceeds 20,000 characters)

Reviewer #1: I have accepted to review of the article "Genetic histories of individuals from multi-faith medieval Sicily" (PONE-D-26-04315), which aims to address a topic of great archaeological and anthropological interest: the population composition and relationships between individuals of different ethnic origins, cultures, and religions in medieval and late-medieval Sicily.
Sicily has long attracted the interest of those who approach archaeo-demographic issues with molecular methodologies, and in fact it is one of the most studied regions in the Mediterranean and Europe. This study bases many of its discussions precisely on these previous bibliographical findings, confirming an extraordinary genetic complexity and the establishment, in some geographical parts of the island, of veritable melting pots "ante litteram," a sign that population fluidity was considerable, not only during the Punic and Roman eras, but well before. The hunter-gatherer Sicily was shaped by the Neolithic movement, partly by the Iranian movement, and later by the Steppe movements. These events set the stage and make it difficult to discuss subsequent contributions from the Near and Middle East.
The article's intent is laudable, and the project's results are likely eagerly awaited.
The article examines a significant number of individuals, spread across a vast array of archaeological sites, some decidedly urban (with complex cultural dynamics—Palermo itself, in different neighborhoods, has different dynamics), and others rural. Dating reveals multigenerational distributions in cemeteries, and things can change significantly in four to eight generations. The geographic distribution of the sites is not representative of the island's complexity and its population and multicultural distribution. The problem is mainly objective: medieval Sicily has often been excavated with unsystematic approaches and studied, both archaeologically and anthropologically, with outdated approaches and models.
It follows, therefore, that, upon closer inspection, the sampling is poorly representative, not only for Christian burials, as the authors themselves admit in their conclusions, but also for Islamic sites or those presumed to be Islamic.
The article thus turns itself into a series of "minimal histories," especially for the more exotic individuals, and this is interesting and will be useful for other and subsequent studies. The minimal histories are important (although I wouldn't be so impressed by African provenances. Movement from the continent in prehistoric, protohistoric, classical, Late A. and medieval times is, in fact, already documented).

Response: Thank you for the kind words. Indeed, our intention was to interpret the genetic data using a "minimal history" perspective and shed some light on stories of the individuals.

We expect the results will be helpful to many other investigations of medieval groups and the history of Mediterranean populations.

From a methodological standpoint, the article is conducted with rigor and with adequate molecular analysis and bioinformatics tools, but it lacks effective resolution in reconstructing the aforementioned minimal histories and generational relationships. A cross-approach with the study of Strontium and Oxygen isotopes would have been very useful, as well as, wanting to focus the work on individuals, an effort at phenotypic characterization for the individuals with the greatest and most effective coverage, would have been intriguing.

Response: Thank you for raising the question regarding strontium and oxygen isotope analyses. We agree that isotopic data represent an important line of evidence, and indeed our team has used such an approach in the previously published Segesta project (Monnereau et al. 2024). Isotopic analysis has also been applied to some archaeological individuals in this current work, however, this component has not been completed on the same timeline. Those methods are led by other members of the wider team and the timing of DNA and unfortunately isotopic data could not be aligned for publication. Therefore, we have kept this manuscript focused on the minimal histories which can be identified from the genetic data alone. We eagerly await learning whether the isotopic data can provide further contexts to the individuals with divergent genetic ancestries.

I found the reading frankly laborious. I therefore recommend a significant revision of the text's prose and figure legends.
I would give more substance to the "Global Perspectives" paragraph, which is confirmatory. I would also rewrite the "Medieval Connections with Sub-Saharan Africa," which has more difficult or misleading periods (Is Ghana not in West Africa?), and I would revise the discussion on Admixture.

Response: We have worked to improve the prose, particularly in the paragraphs titled 'Global Perspectives' and 'Medieval Connections with Sub-Saharan Africa', and about the admixture analysis and discussion as suggested.

I suggest inserting a table in the main text that includes the variety of mitochondrial and Y characterizations by individual, archaeological provenance, and 14C.

Response: We explored including such a table in the main text, but given the large number of archaeological individuals tested, it would unfortunately be too long and complex for a standard paper size. The mitochondrial and Y haplotypes are reported in Table S1 along with the archaeological provenance, radiocarbon date and other DNA statistics.

The authors sometimes use the term "Phoenician," but there is abundant evidence, especially archaeological and now molecular, that the Phoenicians (strictly speaking) have very little to do with it, and I personally recommend avoiding it.

Response: This was a helpful point and we have removed nearly even mention of "Phoencian". We don't mean to reinforce misconceptions about specific groups of people in the past, but we have used the term in one sentence in the Discussion section to be consistent with terminology in another publication. We hope this ensures that our findings can be compared to the other project in an unambiguous way, while still avoiding misunderstandings about the Phoenicians.

I may be mistaken, but some authors appear in the acknowledgements. I would reword the paragraph itself.

Response: Thank you very much for catching this error. We have corrected the list.

The article, despite the above considerations, is original, has useful results, and meets the required criteria. Therefore, the article is acceptable after a thorough review.

Reviewer #2: The manuscript "Genetic histories of individuals from multi-faith medieval Sicily" presents an ancient DNA study of medieval Sicily, a region of major historical and geopolitical importance due to its position at the crossroads in the Mediterranean area. By analyzing 111 individuals, the study addresses a significant gap in the literature by focusing on relatively recent historical periods using ancient genomic methodologies.
Overall, the manuscript is well written, the research question is timely, and the dataset represents a valuable contribution to Mediterranean population history.
I have only a few minor comments:
- line 149: please report additional decimal places where possible. Value expressed as 0.00X are not sufficiently informative

Response: We have updated it.

- Lines 307–308: even if reported elsewhere in the manuscript, please also indicate here the number of individuals included in this analysis.

Response: Thank you for pointing it out, we updated this information.

- Lines 383–384: are you referring here to the individuals who yielded sufficient mitochondrial and/or nuclear data? Please clarify this point to avoid ambiguity

Response: We have clarified this point.

- Line 532: please refer to the Supplementary Materials for further details on the protocols used.

Response: It has been updated.
